# Rational design of multi-epitope vaccine for Chandipura virus using an immunoinformatics approach

**DOI:** 10.1371/journal.pone.0335147

**Published:** 2025-10-23

**Authors:** Ramtin Naderian, Sajjad Ahmad, Mojgan Rahmanian, Shahrzad Aghaamoo, Aryan Rahbar, Omid Pajand, Akram Alizadeh, Shahin Nazarian, Samira Sanami, Majid Eslami

**Affiliations:** 1 Nervous System Stem Cells Research Center, Semnan University of Medical Sciences, Semnan, Iran; 2 Department of Health and Biological Sciences, Abasyn University, Peshawar, Pakistan; 3 Abnormal Uterine Bleeding Research Center, Semnan University of Medical Sciences, Semnan, Iran; 4 Social Determinants of Health Research Center, Semnan University of Medical Sciences, Semnan, Iran; 5 Department of Electrical and Computer Engineering, University of Southern California, Los Angeles, California, United States of America; 6 Department of Bacteriology and Virology, Semnan University of Medical Sciences, Semnan, Iran; Bangladesh Council of Scientific and Industrial Research, BANGLADESH

## Abstract

Chandipura virus (CHPV) is endemic in India, with frequent outbreaks reported. No approved medicines or vaccines exist for CHPV. We aimed to develop a multi-epitope vaccine for CHPV using immunoinformatics approaches. In this study, a multi-epitope vaccine construct was developed by combining 11 CTL epitopes, 2 HTL epitopes, and 1 linear B-cell epitope from glycoprotein (G) with 1 EAAAK linker, 10 AAY linkers, 2 GPGPG linkers, 1 KK linker, and adjuvant (RS-09 peptide). We predicted and optimized the vaccine’s protein structure. Furthermore, the vaccine 3D structure was docked with Toll-like receptor 4 (TLR4) using the Cluspro 2.0 server, and the docked complex was analyzed using molecular dynamics (MD) simulation by the assisted model building with energy refinement (AMBER) v.20 package. The vaccine’s immune simulation profile was determined, and the vaccine sequence was reverse translated and *in silico* cloned into the pET28a (+). The vaccine’s population coverage was 99.79% across the worldwide. The vaccine was soluble, non-allergenic and non-toxic, with high levels of antigenicity. The quality of the vaccine’s 3D structure improved following refining, and the number of residues in the most favoured regions of the Ramachandran plot increased by 94.2%. The molecular docking, with a docking score of −1157 kcal/mol, and MD simulation results revealed a robust interaction and remarkable stability between the vaccine and TLR4. The immune response simulation indicated a decrease in antigen levels and an increase in interferon‐gamma (IFN‐γ) and interleukin-2 (IL-2) concentrations after each injection. *In silico* results indicate that this vaccine possesses significant promise against CHPV; however, laboratory and animal studies are necessary to validate our findings.

## Introduction

Between early June and mid-August 2024, the Ministry of Health and Family Welfare of India (MOHFW) reported 245 cases of acute encephalitis syndrome (AES), resulting in 82 deaths. Chandipura virus (CHPV) was identified in 64 of these cases. CHPV is endemic in India, and previous epidemics have occurred on a constant basis. However, the current outbreak is the largest in the last two decades [1]. The virus was first identified in Chandipura village, Nagpur, India, during an outbreak of feverish illness caused by the dengue and chikungunya viruses in 1965 [2]. It caused outbreaks in Andhra Pradesh (2003) [3,4], Gujarat (2004) [5], Nagpur (2007) [6], and Odisha (2009) [7]. It is the leading cause of acute encephalitis among India’s pediatric population [3].

CHPV is transmitted by sandflies (*Phlebotomus* sp. or *Sergentomyia* sp.) and mosquitos (*Aedes aegypti*) [8]. The disease is clinically defined by a high-grade fever of brief duration, emesis, sensory abnormalities, neurological dysfunction, and widespread seizures, which can quickly progress to convulsions, coma, or death [9]. The diagnosis of CHPV infection relies on a series of laboratory tests performed on clinical specimens. These assays include the identification of CHPV RNA, virus isolation, and the detection of CHPV-specific IgM antibodies. The real-time one-step reverse transcription-polymerase chain reaction (RT-PCR) test is an excellent method for CHPV detection, with several advantages including high sensitivity, rapidity, precision, and repeatability [8].

CHPV is classified within the *Vesiculovirus* genus of the *Rhabdoviridae* family [10]. CHPV has a negative-sense single-stranded RNA about 11 kb that encodes five proteins: nucleocapsid protein (N), phosphoprotein (P), matrix protein (M), glycoprotein (G), and large protein (L) [11]. The many stages of the viral life cycle are made possible by the interaction of these proteins with the machinery of the host cell. These stages include the entry and uncoating of the virus, the transcription and replication of the virus’s genome, and finally the assembly and release of progeny virions [12]. The G-protein is a trimeric trans-membrane glycoprotein that is the only spike protein of CHPV. It is responsible for adsorption, assembly, and budding of the virus, and it also triggers an antibody response, making it a significant antigenic determinant [13].

Vaccination has proven undeniably beneficial in fostering a healthy global population. It has preserved numerous lives, reduced healthcare costs, and enhanced human quality of life. Nonetheless, emerging and reemerging infectious diseases (ERID), characterized by complex life cycles and antigenic diversity, provide substantial challenges to vaccine development [14]. Reverse vaccinology has recently been able to surpass conventional approaches to vaccine development thanks to advancements in genome sequencing and recombinant DNA technology. In order to create the multi-epitope subunit vaccine, several bioinformatics methods are used to analyze the target pathogen’s genetic material in search of potential epitopes. This method is widely used because to its cost-effectiveness and great specificity in inducing humoral and cell-mediated immune responses [15]. The fundamental disadvantage of multi-epitope-based vaccines is their low immunogenicity when used alone, which can be addressed by the addition of adjuvants [16].

The design of multi-epitope vaccines is a burgeoning field that has already attained significance. Vaccines developed through this methodology have demonstrated in vivo efficacy and protective immunity [17–19]. Additionally, several multi-epitope vaccine candidates, including EMD640744, have progressed to phase I clinical trials [20]. Also, Moderna (Spikevax) and Pfizer-BioNTech (Comirnaty) monovalent XBB.1.5 mRNA COVID-19 vaccines are examples of successful U.S. Food and Drug Administration (FDA)-approved mRNA vaccines, both of which have shown significant effectiveness in preventing COVID-19 in different populations [21–24].

**Fig 1:**
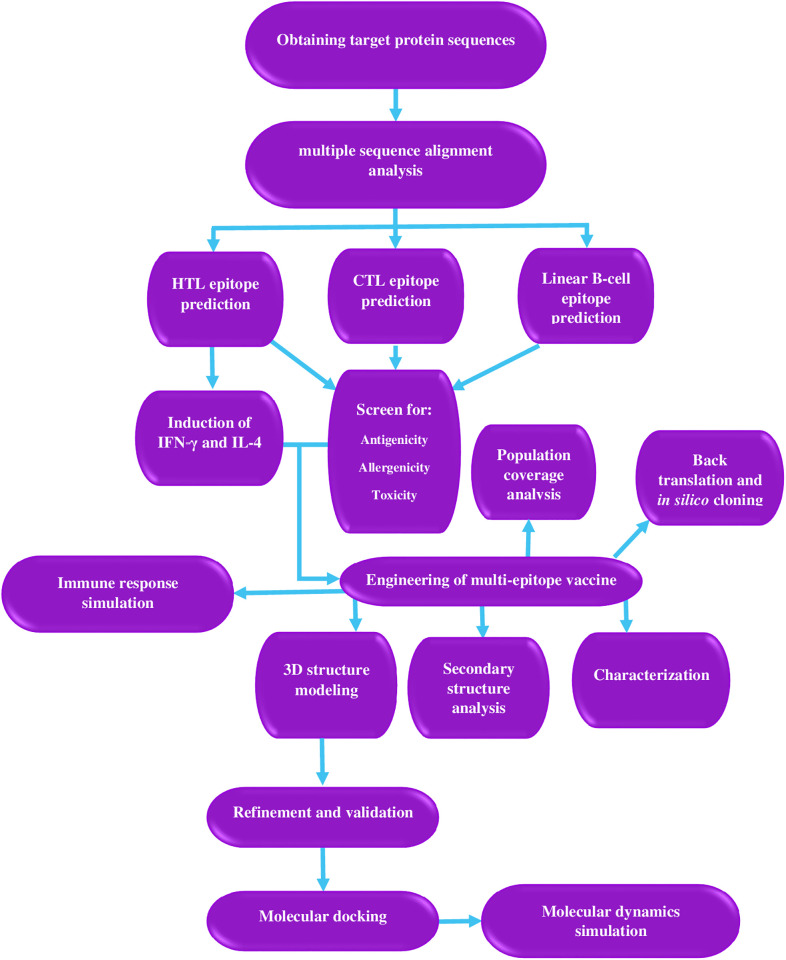
Flowchart of the design of the multi-epitope vaccine in the current study.

The case-fatality ratio for CHPV infection is very high (56–75%), and no specific treatment or vaccine exists [1]. Thus, considering the virus’s global menace, an effective multi-epitope vaccine against CHPV is urgently required. The purpose of this effort is to develop a multi-epitope vaccine against CHPV by combining bioinformatics, computational informatics, and modeling approaches. Fig 1 depicts a flowchart for the methods employed in this study.

## Methods

### Obtaining target protein sequences and multiple sequence alignment analysis

We obtained the G protein sequences of CHPV from the National Center for Biotechnology Information (NCBI) database (https://www.ncbi.nlm.nih.gov/). Partial and irrelevant sequences with ambiguous characters were removed, and the remaining sequences underwent multiple sequence alignment (MSA) using Clustal Omega tool (https://www.ebi.ac.uk/jdispatcher/msa/clustalo) with the default settings [25].

### Epitope prediction

The identification of cytotoxic T lymphocyte (CTL) epitopes is critical for vaccine development and understanding of T-cell activation processes, and it has led to advances in cancer immunotherapy and a variety of infectious diseases [26]. The MHC I binding prediction module from the Immune Epitope Database (IEDB) (http://tools.iedb.org/mhci/) was used to predict CTL epitopes. CTL epitopes for the HLA allele reference set were predicted using employing the NetMHCpan 4.1 EL (recommended epitope predictor-2023.09) method, while other parameters were kept at their default values. NetMHCpan 4.1 includes independent binding (BA) and elution (EL) predictors. BA predictions assess a peptide’s binding capacity to an MHC molecule; EL predictions consider the potential that the peptide will be naturally processed and presented, boosting the likelihood of identification as a T cell epitope [27]. Helper T lymphocytes (HTLs) are essential for establishing both humoral and cellular immune responses. The HTL epitopes induce a CD4^+^ helper response, which is essential for the generation of protective CD8^+^ T-cell memory and the stimulation of B-cells to produce antibodies [28,29]. To predict HTL epitopes, we employed the IEDB’s MHC-II binding prediction module (http://tools.iedb.org/mhcii/). HTL epitopes with binding affinity to the full HLA reference set were predicted using the NetMHCII pan 4.1 EL (recommended epitope predictor-2023.09) method [30]. Each predicted T-cell epitope is assigned a percentile rank, and a low percentile rank indicates high affinity. T-cell epitopes having a percentile rank ≤1 was considered for further analysis [31,32]. Linear B-cell epitopes play a significant role in the development of peptide vaccines and can easily be used to replace antigens for antibody production [33]. Based on the Bepipred Linear Epitope Prediction 2.0 method, we predicted linear B-cell epitopes using the IEDB’s antibody epitope prediction module (http://tools.iedb.org/bcell/). BepiPred predicts the location of linear B-cell epitopes by combining a hidden Markov model with a propensity scale approach [34]. Among the predicted linear B-cell epitopes, those having a length of 6 amino acids or greater were chosen for further investigation.

### Evaluation of epitopes

The antigenicity, allergenicity, and toxicity properties of the suitable predicted epitopes from the preceding stage were assessed by the VaxiJen v2.0 (https://www.ddg-pharmfac.net/vaxijen/VaxiJen/VaxiJen.html), AllerTOP v. 2.0 (https://www.ddg-pharmfac.net/AllerTOP/), and CSM-Toxin (https://biosig.lab.uq.edu.au/csm_toxin/) servers. The VaxiJen is the first server to predict protective antigens without requiring alignment. The purpose of its development was to enable the classification of antigens entirely based on the physicochemical properties of proteins, without the need for sequence alignment [35–37]. To predict antigenicity via this server, the antigenicity threshold for epitopes was set to 0.4 (default), and “virus” was selected as the target organism. CSM-Toxin is an advanced *in silico* protein toxicity classifier that uses only the protein’s primary sequence. The approach encodes protein sequence information using a deep learning natural language model to comprehend the “biological” language, in which residues are considered as words and protein sequences as sentences. The CSM-Toxin approach successfully identifies potentially toxic peptides and proteins; it has achieved a MCC values of up to 0.66 in both cross-validation and multiple non-redundant blind tests [38]. The AllerTOP v. 2.0 server uses auto cross covariance (ACC) to transform protein sequences into uniform equal-length vectors. The proteins are categorized using the k-nearest neighbor algorithm (kNN, k = 1) with a training set of 2427 known allergens from various species and 2427 non-allergens [39]. In addition to the considering criteria, the HTL epitopes were assessed for their ability to induce interferon‐gamma (IFN‐γ) and interleukin-4 (IL-4) using the IFNepitope server (http://crdd.osdd.net/raghava/ifnepitope/design.php) and IL4pred server (http://crdd.osdd.net/raghava/il4pred/design.php), respectively. The IFNepitope server’s prediction was based on the IFN-γ vs. non-IFN-γ model and the Hybrid (Motif + SVM) approach [40]. The IL4pred server used a hybrid (SVM + Motif) prediction method, with an SVM threshold of 0.2 [41]. IFN-γ plays a vital role in activating both the adaptive and innate immune systems, as well as inhibiting viral replication [42]. IL-4 directs the development of TH2, which leads to the production of IgE [43].

### Engineering of multi-epitope vaccine

To prevent the repetition of similar sequence in the multi-epitope vaccine’s structure, we excluded epitopes that had their whole sequence present in other selected epitopes. The remaining epitopes were then included in the final structure of the multi-epitope vaccine. Linkers are essential in the design of the vaccine to mimic the immunogen’s ability to work independently and generate larger levels of antibodies compared to a single immunogen [44]. In this study, the Ala-Ala-Tyr (AAY), Gly-Pro-Gly-Pro-Gly (GPGPG), and bi-lysine (KK) linkers were employed to connect selected CTL, HTL, and linear B-cell epitopes, respectively. The AAY linkers enhance the presentation of epitopes and decrease the number of junctional epitopes [45,46]. The GPGPG linker improves the construct’s solubility and also gives adjacent domains freedom to act, easy access, and flexibility [47]. The KK linker helps to preserve the independent immunogenic activities of the construct’s epitopes [48,49]. To increase immunogenicity, an RS-09 peptide (TLR4 agonist) sequence was attached to the N-terminus of the multi-epitope vaccine via the linker EAAAK. RS-09 is known as an adjuvant due to its capacity to elicit significant immunological activation and enhance antibody production. Moreover, the involvement of RS-09 facilitates the concurrent activation of CTL epitopes, resulting in enhanced immunological stimulation. The use of synthetic adjuvants (RS-09) is a safer approach relative to traditional adjuvants, providing enhanced immunity and superior efficacy compared to traditional adjuvants [50,51]. The EAAAK linker is a stiff linker that, due to its capacity to form helix, improves flexibility, facilitates folding, and increases the stability of the multi-epitope vaccine structure [52]. Finally, a His-tag (also called 6xHis-tag) was attached to the C-terminus of the vaccine construct to aid protein purification while maintaining the functioning of the fusion proteins [53].

### Population coverage analysis

The high frequency of HLA alleles associated with vaccine constituent epitopes gives a clear indication of vaccine efficacy in different geographic regions [54]. The population coverage analysis was carried out using the IEDB’s population coverage tool (http://tools.iedb.org/population/). The CTL and HTL epitopes, which are used in vaccine development, were evaluated both separately and in combination to determine their population coverage throughout 16 different geographical areas, India, and world [55].

### Characterization of multi-epitope vaccine

The antigenicity level of the multi-epitope vaccine was predicted using the VaxiJen v2.0 server with a threshold value of 0.4, and the ANTIGENpro server (https://scratch.proteomics.ics.uci.edu/). ANTIGENpro is a predictor of protein antigenicity that is based on sequence analysis, does not require alignment, and is not limited to certain pathogens [56]. The AllerTOP v. 2.0 and AllergenFP v.1.0 (https://ddg-pharmfac.net/AllergenFP/) servers were employed to assess the allergenicity of the vaccine candidate. The AllergenFP v.1.0 server employs an algorithm that characterizes the amino acids in the protein sequence using five descriptors, and then converts the strings into uniform vectors by the ACC transformation [57]. The toxicity of the multi-epitope vaccine was assessed using the CSM-Toxin server. The proposed vaccine’s solubility was evaluated using the SOLpro (https://scratch.proteomics.ics.uci.edu/) and Protein-Sol (https://protein-sol.manchester.ac.uk/) servers. SOLpro utilizes a two-stage SVM architecture to predict the solubility of a protein when overexpressed in *Escherichia coli* (*E. coli*). This prediction is made by considering several representations of the protein’s primary sequence [58]. Protein-Sol is a web server for predicting protein solubility, and its algorithm predicts the solubility of protein sequences based on available data for *E. coli* protein solubility in an expression system. In this server, the population mean for the experimental data set (PopAvrSol) is 0.45, therefore a predicted solubility value greater than 0.45 indicates higher solubility than the average soluble *E.coli* protein [59]. Furthermore, the Expasy ProtParam tool (https://web.expasy.org/protparam/) was used to estimate the physicochemical parameters of the multi-epitope vaccine. This tool predicts a variety of data, including the molecular weight, theoretical pI, total number of negatively and positively charged residues, formula, total number of atoms, half-life, instability index, aliphatic index, and grand average of hydropathicity (GRAVY) [60]. The molecular weight of a protein is the sum of the molecular weights of its constituent amino acids, along with the weights derived from posttranslational modifications [61]. The pI is the pH at which the net charge of a protein becomes zero [62]. The half-life is a prediction of how long it will take for half of the protein in a cell disappear once it has been synthesized [63]. The instability index estimates the stability of a protein in a test tube [64]. The protein’s aliphatic index is defined as the relative volume occupied by aliphatic side chains [65]. The GRAVY value for a peptide or protein is determined by summing the hydropathy values of all amino acids and dividing by the total number of residues in the sequence [66].

### Secondary structure analysis

The PDBsum server (https://www.ebi.ac.uk/thornton-srv/databases/pdbsum/) was employed to determine the secondary structure of the multi-epitope vaccine. This server provides access to the structural information of Protein Data Bank (PDB) entries. The server includes protein secondary structure, interactions between proteins, ligands, and DNA, PROCHECK structural quality checks, and a multitude of other image-based analyses [67].

### 3D structure modeling, refinement, and validation

The Robetta server (https://robetta.bakerlab.org/) was employed to predict the 3D model structure of the vaccine construct. The Robetta server provides automated tools for predicting and evaluating protein structures. This server analyzes the given sequences to identify probable domains and constructs structural models using either comparative modeling or de novo structure prediction techniques for predicting the structure. When a protein with a known structure is found to be a highly similar match using Basic Local Alignment Search Tool (BLAST), Position-Specific Iterative BLAST (PSI–BLAST), Fold and Function Assignment System (FFAS), or 3D-Jury, it is used as a template for comparative modeling. If there is no match, structural predictions are made using the de novo Rosetta fragment insertion approach [68]. To get the predicted 3D structure closer to the proteins’ native structure, we refined the vaccine’s 3D structure using Galaxy Refine server (https://galaxy.seoklab.org/cgi-bin/submit.cgi?type=REFINE). The GalaxyRefine web server employs a refining process that has been thoroughly tested in CASP10. The method uses molecular dynamics (MD) modeling to restore side chains, repackage them, and finally relax the overall structure. The CASP10 evaluation revealed that this strategy had the greatest impact on the quality of local structures. This method can improve the average quality of both global and local structures [69]. The PROCHECK tool from the SAVES v6.0 server (https://saves.mbi.ucla.edu/) and the ProSA-web server (https://prosa.services.came.sbg.ac.at/prosa.php) were used to evaluate the quality of the vaccine’s initial and refined models. The PROCHECK performs a thorough assessment on the stereochemistry of a protein structure. It produces a number of PostScript charts and a detailed residue-by-residue listing. These provide an assessment of the structure’s overall quality when compared to well-refined structures of the same resolution, as well as highlighting places that may require additional examination [70,71]. The ProSA-web server calculates and presents a Z-score for a given input structure. The Z-score measures the model’s overall quality and is displayed in a plot that includes the Z-scores of all experimentally determined protein chains. Different colors indicate distinct groupings of structures in this plot, which were derived from various sources such as X-ray and NMR. This server be utilized to verify if the Z-score of the given structure falls into the range of scores often observed for native proteins of comparable size [72,73].

### Prediction of discontinuous B‑cell epitopes

IEDB’s ElliPro tool (http://tools.iedb.org/ellipro/) was used to predict the discontinuous B cell epitopes in the vaccine’s refined 3D structure. The ElliPro provides a score to each predicted epitope based on the average Protrusion Index (PI) value across its residues. The prediction method was based on default parameters, with a minimum score of 0.5 and maximum distance of 6 Å [74].

### Molecular docking between vaccine and TLR4

The Cluspro 2.0 server (https://cluspro.org/login.php) was used to estimate the affinity of interaction between vaccine’s refined 3D structure and Toll-like receptor 4 (TLR4) [75]. The PDB file for TLR4 (PDB ID: 4G8A) was retrieved from the RCSB Protein Data Bank (PDB), and the extra bound ligands and water were removed from its structure using the ChimeraX 1.8 program. The TLR4 as receptor and the vaccine’s refined 3D structure as ligand were then sent to the ClusPro 2.0 server to perform molecular docking. Subsequently, the interaction between the vaccine and the TLR4 was analyzed using the PDBsum server (http://www.ebi.ac.uk/thornton-srv/databases/pdbsum/) [67].

### Molecular dynamics simulation analysis

The selected docked complex was subjected to MD simulation, a method for studying biological and chemical systems at the atomic level on time scales ranging from femtoseconds to milliseconds [76]. The assisted model building with energy refinement (AMBER) v.20 package was employed for conducting simulations and subsequent analyses using its various modules [77]. The antechamber program was utilized for the preparation of the complexes [78]. The Leap program was used to submerge complexes in the TIP3P solvation box. The ff14SB force field was employed to clarify the system’s intermolecular interactions. To neutralize the system’s overall charges, chloride and sodium ions were introduced to the protein surface at the optimum quantities. The energy minimization parameters were optimized using the steepest descent integrator with 5000 steps. The system was then gradually heated to 300 K, with temperature controlled throughout the experiment using the Langevin algorithm. The SHAKE approach was employed to constrain all hydrogen bonds (H-bonds). Using the NPT ensemble, the system was brought into equilibrium at constant pressure. The final step was to run the main 250 nanoseconds (ns) MD simulation on the prepared complex.

### Binding free energy calculation

The molecular mechanics Poisson–Boltzmann surface area (MM/PBSA) and molecular mechanics generalized Born surface area (MM/GBSA) approaches were used to calculate the binding free energies of the selected docked complex. This was accomplished via AMBER’s MMPBSA.py. 1000 frames were picked at regular intervals from simulated trajectories for the determination of binding energy.

### Immune response simulation of the vaccine

The C-IMMSIM server (https://kraken.iac.rm.cnr.it/C-IMMSIM/index.php) was utilized to model the host immune response elicited by the injection of the vaccine candidate. The C-IMMSIM server uses a Position Specific Scoring Matrix (PSSM) generated from machine learning techniques to offer a strong agent-based model for reliably predicting immunological interactions. The C-IMMSIM server concurrently simulates three compartments: bone marrow, thymus, and a tertiary lymphatic organ (e.g., a lymph node), reflecting three distinct anatomical areas in mammals [79]. In accordance with the recommendation for a minimum four-week interval between vaccine doses, three doses were provided during the simulation, with a four-week interval between each [80]. In this study, the time steps were set at 1, 84, and 168, with each interval corresponding to 8 hours in real life. The simulation steps were set for 1050 (equivalent to 350 days), with all other parameters remaining at their default values.

### Back translation and *in silico* cloning

To enhance vaccine protein production with appropriate post-translational modifications [81], the vaccine protein sequence was submitted to the Gene Infinity server (https://www.geneinfinity.org/sms/sms_backtranslation.html) for back translation and codon optimization in the *E. coli* host strain. The GenScript server (https://www.genscript.com/tools/rare-codon-analysis) then measured the Codon Adaptation Index (CAI) and the GC content of the cDNA sequence. The sequences of the restriction enzymes *XhoI* (5’-CTCGAG-3’) and *XbaI* (5’-TCTAGA-3’) were attached to the 5’ and 3’ ends of the cDNA sequence, respectively; ultimately, this resultant sequence was inserted into the pET-28a (+) vector using SnapGene v7.2 software (https://www.snapgene.com/free-trial).

### Ethics statement

The ethical committee of Semnan University of Medical Sciences approved this study with the number: IR.SEMUMS.REC.1403.141.

## Results

### Obtaining target protein sequences and multiple sequence alignment analysis

A total of 32 G protein sequences in FASTA format were collected from the NCBI database, from which 26 appropriate sequences were selected after removing incomplete sequences. The MSA was conducted with Clustal Omega software to determine the conserved sections of the target proteins (S1 Data). The selection of conserved regions was based on two criteria: the absence of gaps in the protein sequence and the highest conservation of amino acids.

### Epitope prediction and screening

The IEDB predicted 62 CTL epitopes (percentile rank ≤1), 9 HTL epitopes (percentile rank ≤1), and 8 linear B-cell epitopes (with a minimum length of 6 amino acids) from the G protein. After evaluating the predicted epitopes for their antigenicity, allergenicity, toxicity, and ability to produce IFN-γ and IL-4, 12 CTL epitopes (S1 Table), 3 HTL epitopes (S2 Table), and 1 linear B-cell epitope (S3 Table) were selected to be the most optimal epitopes.

### Engineering of multi-epitope vaccine

**Fig 2:**
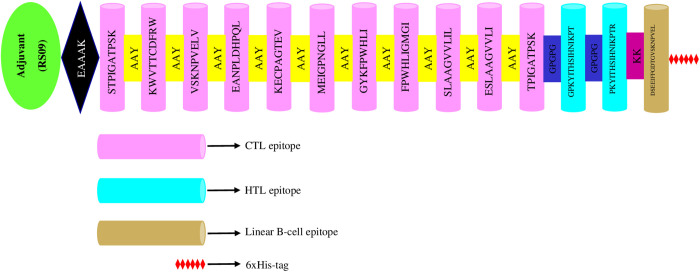
The graphic illustration of the final configuration of the multi-epitope vaccine.

The multi-epitope vaccine was developed by coupling 11 CTL epitopes, 2 HTL epitopes, and 1 linear B-cell epitope with 1 adjuvant sequence (APPHALS), 1 EAAAK linker, 10 AAY linkers, 2 GPGPG linkers, 1 KK linker, and 1 His-tag (Fig 2). The designed multi-epitope vaccine consists of 214 amino acids.

### Population coverage analysis

**Fig 3:**
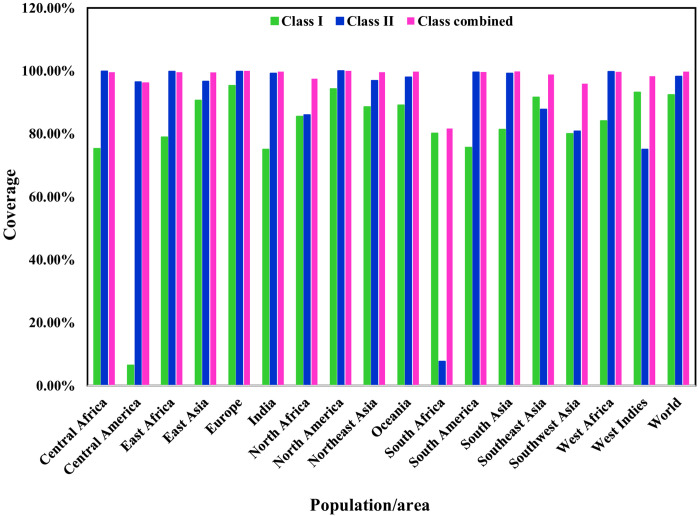
Population coverage analysis of the selected T-cell epitopes based on their corresponding HLA alleles.

During the investigation of population coverage, it was found that the CTL and HTL epitopes individually covered around 75.05% and 99.21% of the India population, respectively; however, when these epitopes were used in combination, the population coverage increased to 99.77%. The population coverage of the selected CTL epitopes is highest in Europe (95.34%) and lowest in Central America (6.44%). The selected HTL epitopes has the highest population coverage in North America (99.99%), whereas it has the lowest population in South Africa (7.65%). In all geographic regions except South Africa (81.68%), the selected CTL and HTL epitopes in combination mode had population coverage more than 90%. Fig 3 and S4, S5 and S6 Tables show detailed CTL, HTL, and combined CTL and HTL epitope population coverage by continent.

### Characterization of multi-epitope vaccine

**Fig 4:**
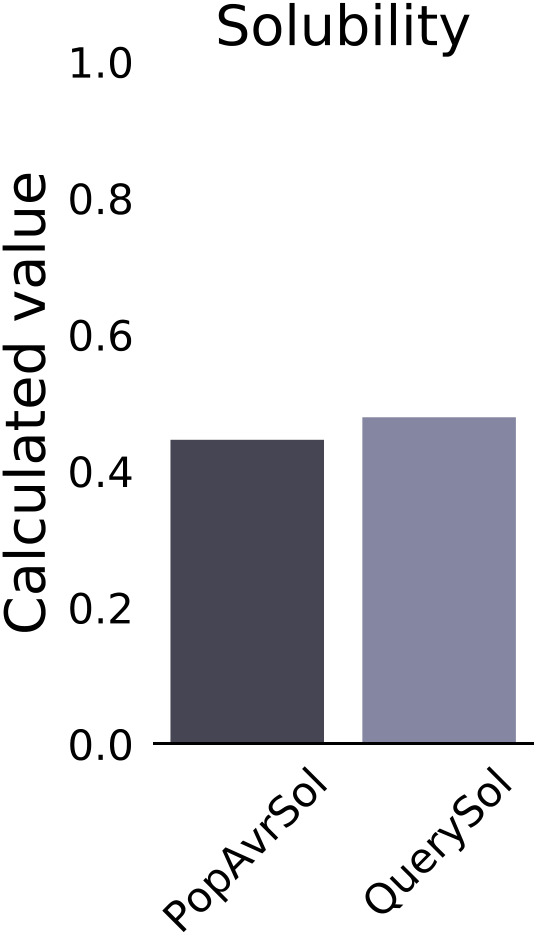
The solubility plot of the multi-epitope vaccine. The vaccine’s solubility is predicted to be 0.479.

The vaccine’s antigenicity score was predicted by the VaxiJen v2.0 server as 0.6769 (> 0.4), while the ANTIGENpro server estimated it as 0.945425. The multi-epitope vaccine was predicted to be non-allergen by both AllerTOP v. 2.0 and AllergenFP v.1.0 servers. The CSM-Toxin server indicated that the vaccine is non-toxic in nature. The SOLpro server indicated a solobility score of 0.967913, while the Protein-Sol server reported a solobility score of 0.479 (Fig 4), with a default threshold of 0.4. The Expasy ProtParam tool predicted the physicochemical parameters of the proposed vaccine construct. These parameters are presented in Table 1.

**Table 1:** The physicochemical parameters of the multi-epitope vaccine.

Parameters	Assessment
Molecular weight	22.79 kDa
Theoretical pI	8.45
Total number of negatively charged residues (Asp + Glu)	14
Total number of positively charged residues (Arg + Lys)	16
Formula	C_1054_H_1589_N_273_O_286_S4
Total number of atoms	3206
Half-life	4.4 h (mammalian reticulocytes, *in vitro*) >20 h (yeast, *in vivo*) >10 h (*E. coli*, *in vivo*)
Instability index	36.29
Aliphatic index	83.18
GRAVY	−0.062

### Secondary structure analysis

**Fig 5:**
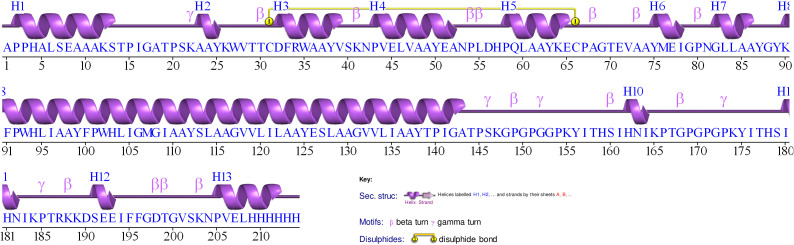
A graphical illustration of the secondary structure of the vaccine predicted by the PDBsum server.

According to the PDBsum server, the proposed vaccine contains 13 helices, 15 helix-helix interacs, 14 beta turns, 5 gamma turns, and one disulphide bond (Fig 5).

### 3D structure modeling, refinement, and validation

**Fig 6:**
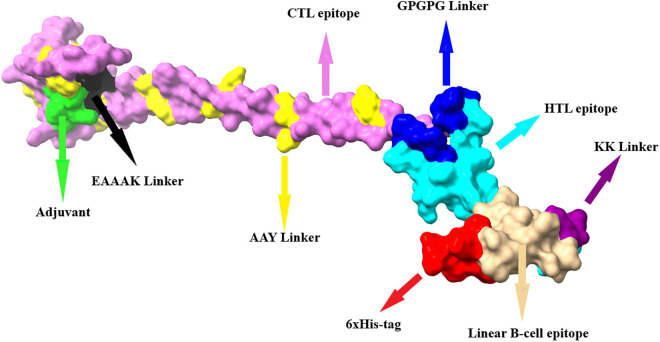
The refined three-dimensional structure of the multi-epitope vaccine.

**Fig 7:**
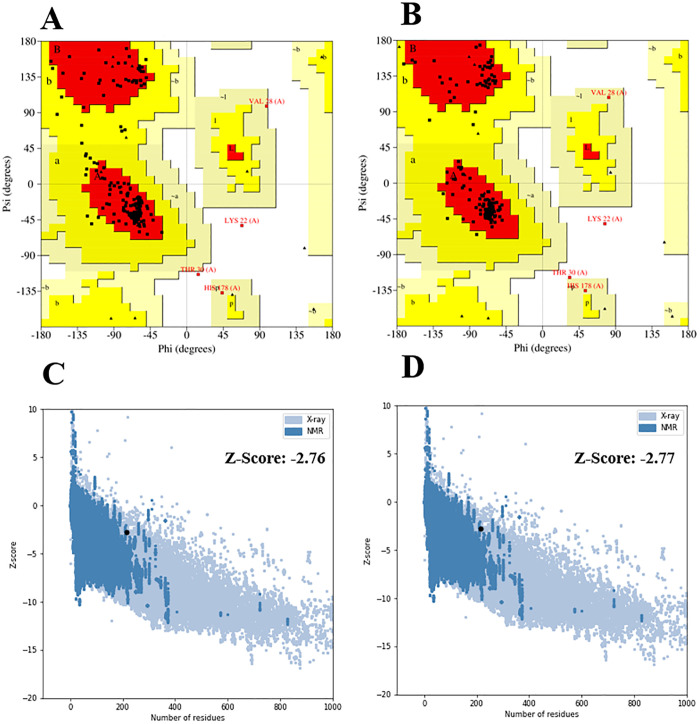
Validation of the vaccine candidate’s initial and refined 3D structural models. The Ramachandran plot reveals that the number of residues located in the most favoured regions increased from 87.7% in the initial model (A) to 994.2%in the refined model (B). The ProSA Z-score of the initial model was −2.76 (C), whereas that of the refined model was −2.77 (D).

The Robetta server generated five 3D structures for the multi-epitope vaccine. Out of the 5 models predicted by the server, model 1 was chosen as the best model after evaluating it using the Ramachandran plot. The chosen model was refined using the GalaxyRefine server. The GalaxyRefine server produced five refined models with varying quality assessment criteria, such as GDT-HA, RMSD, MolProbity, Clash score, Poor rotamers, and Rama favored (Table 2). The model 2 was chosen for further analyses due to its high GDT-HA (0.9755) and Rama favored (97.2) values, as well as low RMSD (0.332), MolProbity (1.544), Clash score (4.3), and Poor Rotamers (0.0) values (Fig 6). The PROCHECK tool and the ProSA-web server were utilized to assess the comparative quality of the vaccine’s 3D structure pre- and post-refinement. The Ramachandran plot analysis revealed that in the initial model, 87.7% of residues were located in the most favoured regions, and in the refined model, this value increased to 94.2%; in the initial model, the amount of residues in the additional allowed regions was 9.9%, while in the refined model, this region had 3.5% residues; in each of the generously allowed and disallowed regions, 1.2% residues were found in both models (Fig 7A and 7B). The ProSA Z-scores for the initial and refined models were −2.76 and −2.77, respectively (Fig 7C and7D).

**Table 2:** Quality evaluation parameters of models refined by the GalaxyRefine server.

Model	GDT-HA	RMSD	MolProbity	Clash score	Poor rotamers	Rama favored
Initial	1.0000	0.000	1.461	2.9	0.0	94.3
MODEL 1	0.9731	0.343	1.531	5.9	0.6	96.7
MODEL 2	0.9755	0.332	1.544	4.3	0.0	97.2
MODEL 3	0.9696	0.346	1.422	7.2	0.6	96.7
MODEL 4	0.9614	0.370	1.516	5.0	0.6	96.2
MODEL 5	0.9720	0.339	1.585	6.8	0.0	96.7

### Prediction of discontinuous B‑cell epitopes

**Fig 8:**
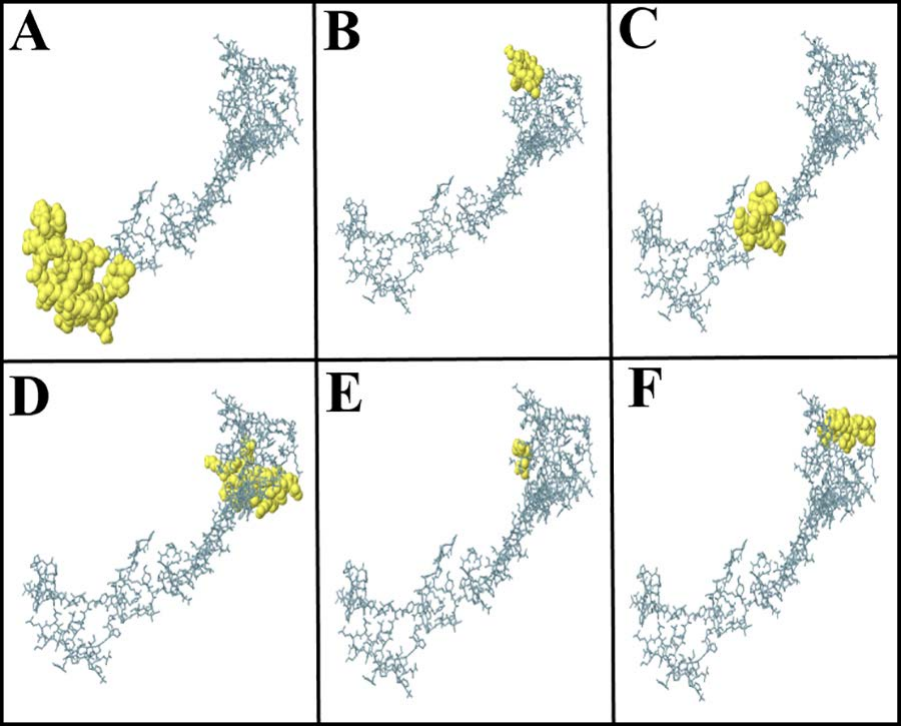
Visualization of discontinuous B-cell epitopes on the vaccine’s 3D structure. (A) Discontinuous B-cell epitope with 39 residues and a score of 0.806, (B) discontinuous B-cell epitope with 13 residues and a score of 0.782, (C) discontinuous B-cell epitope with 13 residues and a score of 0.736, (D) discontinuous B-cell epitope with 22 residues and a score of 0.659, (E) discontinuous B-cell epitope with 3 residues and a score of 0.592, and (F) discontinuous B-cell epitope with 13 residues and a score of 0.579.

The IEDB ElliPro tool identified 6 discontinuous B-cell epitopes in the vaccine’s three-dimensional structure, as shown in Fig 8. The scores of these epitopes ranged from 0.579 to 0.806, and their size ranged from 3 to 39 residues (Table 3).

**Table 3:** List of discontinuous epitopes, with their scores and residue counts.

No.	Residues	Number of residues	Score
1	A:I176, A:T177, A:H178, A:S179, A:I180, A:H181, A:N182, A:I183, A:K184, A:P185, A:T186, A:R187, A:K188, A:K189, A:D190, A:S191, A:E192, A:E193, A:I194, A:F195, A:F196, A:G197, A:D198, A:T199, A:G200, A:V201, A:S202, A:K203, A:N204, A:P205, A:V206, A:E207, A:L208, A:H209, A:H210, A:H211, A:H212, A:H213, A:H214	39	0.806
2	A:T14, A:P15, A:I16, A:G17, A:A18, A:T19, A:P20, A:S21, A:K22, A:A23, A:A24, A:Y25, A:K26	13	0.782
3	A:T144, A:P145, A:S146, A:K147, A:G148, A:P149, A:G150, A:P151, A:G152, A:G153, A:P154, A:K155, A:Y156	13	0.736
4	A:A36, A:A37, A:S40, A:K41, A:N42, A:P43, A:V44, A:E45, A:L46, A:V47, A:A48, A:A49, A:Y50, A:E51, A:A52, A:N53, A:P54, A:L55, A:D56, A:H57, A:I78, A:G79	22	0.659
5	A:A1, A:P2, A:P3	3	0.592
6	A:W27, A:V28, A:T29, A:T30, A:C31, A:F33, A:C66, A:P67, A:A68, A:G69, A:T70, A:E71, A:V72	13	0.579

### Molecular docking between vaccine and TLR4

**Fig 9:**
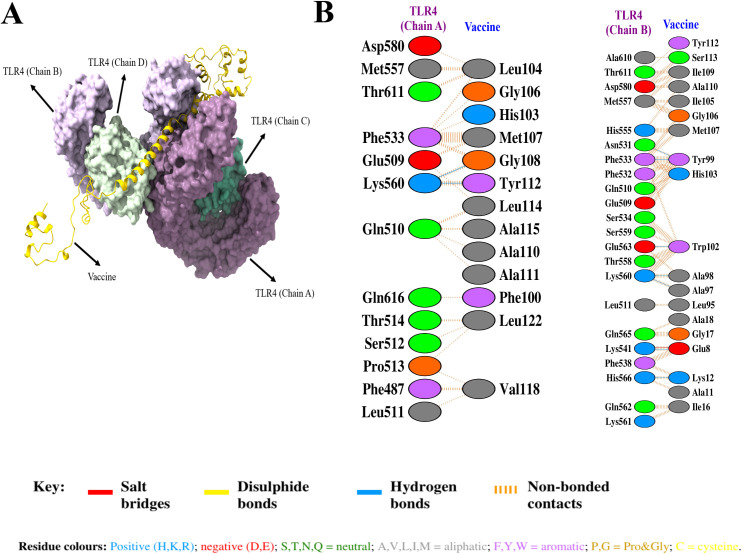
The molecular docking of the vaccine with TLR4. (A) Illustration of the docked complex (vaccine-TLR4). The cartoon model represents the vaccine, whereas the surface model represents TLR4. (B) Interacting residues between the vaccine and TLR4 in the docked complex.

Molecular docking between the vaccine and TLR4 was performed using the ClusPro 2.0 server, yielding 30 docked complexes (S7 Table). The server ranked the constructed complexes based on their energy scores. Cluster 0, with the most members (53) and the most negative energy score (−1157 kcal/mol), was chosen (Fig 9A). Furthermore, interaction residues between the vaccine and TLR4 were visualized using the PDBsum server (Fig 9B). The vaccine-TLR4 interaction involved two H-bonds and 61 non-bonded contacts between 13 vaccine residues and 13 residues of TLR4’s chain A. In addition, there was 1 salt bridge, 7 H-bonds, and 149 non-bonded contacts between 19 vaccine residues and 22 TLR4 chain B residues. The residue pairs involved in the establishment of H-bonds between vaccine and protein are listed in S8 Table.

### Molecular dynamics simulation analysis

**Fig 10:**
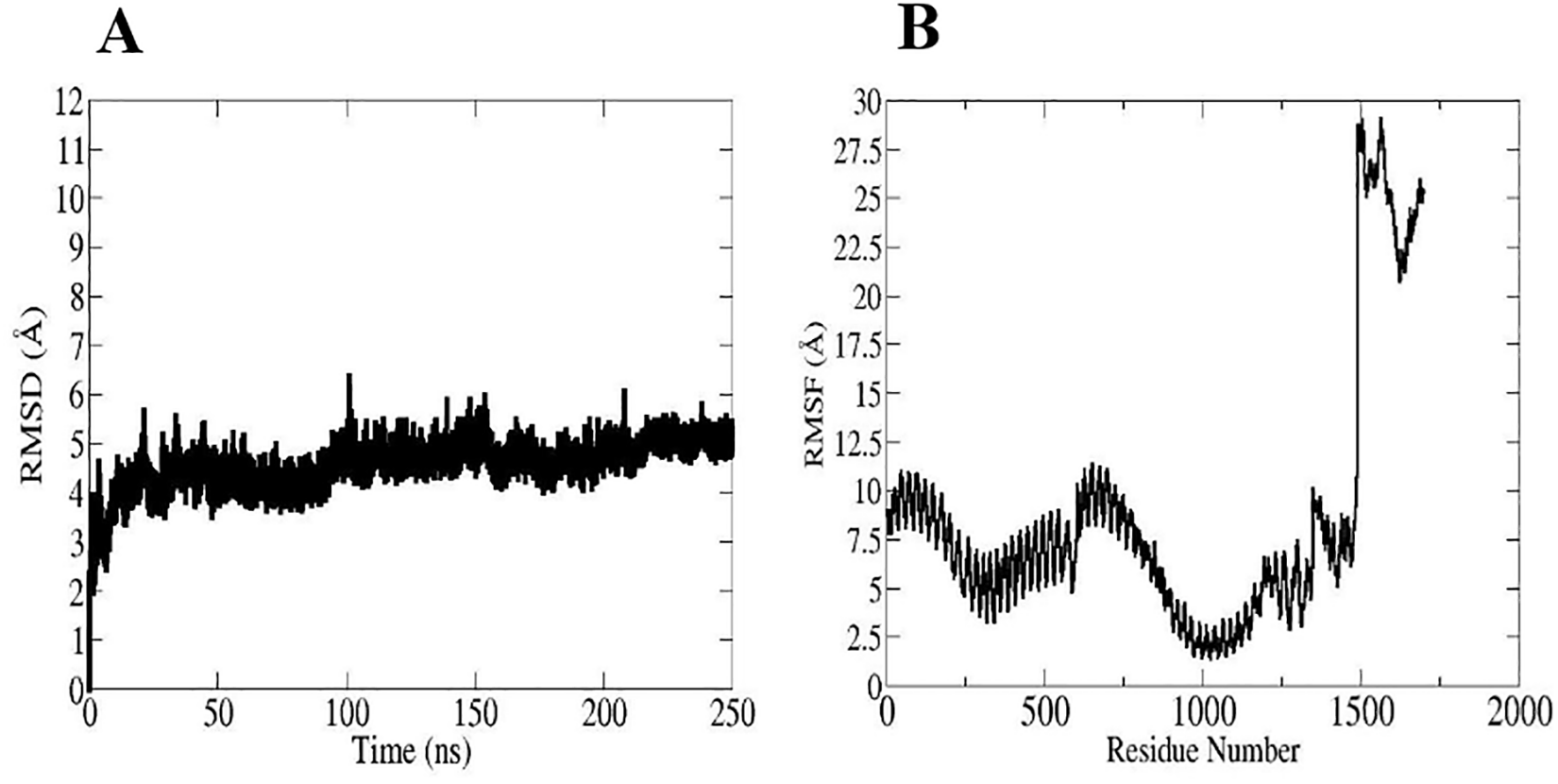
The MD simulation result for the vaccine-TLR4 complex. (A) RMSD plot of the vaccine-TLR4 complex. (B) RMSF plot of the vaccine-TLR4 complex.

Our post-simulation studies included parameter evaluations of root mean square deviation (RMSD) and root mean square fluctuation (RMSF). RMSD is a critical quantifiable parameter for receptor-ligand complex stability [82]. The RMSD plot of the vaccine-TLR4 complex revealed an upward trend in the first 25 ns of the simulation period, followed by oscillations at different time intervals, with a maximum value of 6.4 Å at 100 ns. The RMSD value indicated no significant alteration after 220 ns, and the system remained stable (Fig 10A). The RMSF profile offers useful knowledge into the flexibility of various protein regions, aiding in the comprehension of the protein’s function and its interactions with other molecules [83]. The RMSF plot of the vaccine-TLR4 showed that most of the residues are highly flexible. The vaccine residues (1500–1714) show the most variance, with RMSF values ranging from 20.5 to 28.75 Å (Fig 10B).

### Binding free energy calculation

The binding free energies of the chosen docked complex were computed utilizing the MM/PBSA and MM/GBSA methods. According to the MM/PBSA analysis, the vaccine-TLR4 complex has a total binding free energy of −342.68 kcal/mol. Furthermore, the total binding free energy in the MM/GBSA was determined to be −345.78 kcal/mol for the vaccine-TLR4 complex. The various contributions to the total binding free energy in both approaches show that the formation of the vaccine-TLR4 complex is mostly influenced by the Van der Waals energy interaction (Table 4).

**Table 4:** Binding free energy values for the vaccine-TLR4 complex.

Energy components	Energy value (kcal/mol)
**MMGBSA**	
Van der Waals Energy	−321.0
Electrostatic Energy	−55.32
Gas Phase Energy	−376.32
Solvation Energy	33.64
Net energy	−342.68
**MMPBSA**	
Van der Waals Energy	−321.0
Electrostatic Energy	−55.32
Gas Phase Energy	−376.32
Solvation Energy	30.54
Net energy	−345.78

### Immune response simulation of the vaccine

**Fig 11:**
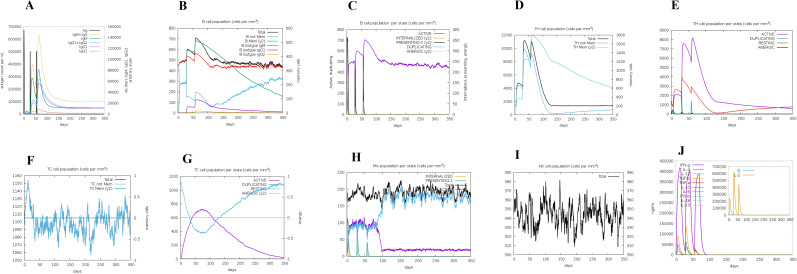
Predicted graphs of the immunological response following the administration of three doses of the proposed vaccine. (A) Antibody and antigen concentrations. (B) B-cells population. (C) B-cells population per state. (D) TH cell population. (E) TH cell population per state. (F) TC cell population. (G) TC cell population per state. (H) Macrophage cells population per state. (I) NK cell population. (J) Induced cytokine levels.

The C-IMMSIM server simulated the immune response induced by the vaccine after three injections. The immune response demonstrated increased concentrations of IgM + IgG, IgM, IgG1 + IgG2, IgG1, and IgG2 antibodies along with a reduction in antigen levels. The second and third responses exhibited superior quality compared to the initial response. The maximum antibody titer rose with the frequency of injections and subsequently diminished after each injection, until attaining a steady level in the body (Fig 11A). An evident rise was observed in the total number of B-cells, B isotype (IgM), memory B cells, and active B cells subsequent to the second and third vaccine injections (Fig 11B and 11C). Following each immunization, there was an increase in the overall number of memory T-helper (TH) cells and active TH cells (Fig 11D and 11E). Similarly, the immunological simulation demonstrated that vaccine administration led to an augmentation in T-cytotoxic (TC) cell populations (Fig 11F and 11G). The immunization stimulates the innate immune system, as demonstrated by the activation and proliferation of macrophages and natural killer (NK) cells (Fig 11H and 11I). The concentration of IFN-γ increased significantly after each injection, as did the amount of cytokines such as TGF-β, IL-10, IL-12, and IL-2. However, the second injection produced more IL-2 and TGF-β than the first and third (Fig 11J).

### Back translation and *in silico* cloning

**Fig 12:**
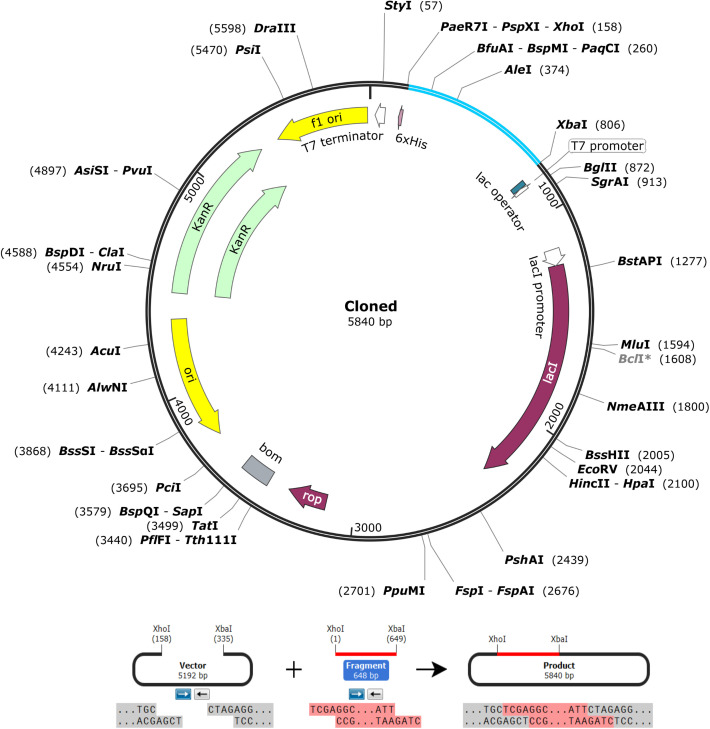
*In silico* cloning of the vaccine cDNA sequence (blue) between the *XhoI* (158) and *XbaI* (806) restriction sites of the pET-28a (+) expression vector (black).

The protein sequence of the vaccine was reverse translated into a 642 bp sequence utilizing the Gene Infinity server. The GenScript server reported the GC content of the optimized sequence as 58% and the CIA value as 1.0. The DNA sequence was then cloned into the pET-28a (+) vector using SnapGene v7.2 software between the restriction enzyme cutting sites *XhoI* (158) and *XbaI* (806), which produced a recombinant plasmid of 5840 bp in length (Fig 12).

## Discussion

CHPV, which is prevalent in tropical and subtropical climates, represents a significant threat to public health across the Indian subcontinent. With increased travel and globalization, viruses are no longer confined to national borders. Furthermore, the identification of CHPV in sandflies on the African continent indicates a substantial risk of its spread, potentially leading to an epidemic in other regions worldwide. In light of these warnings, it is crucial to fully understand CHPV biology and pursue the development of antiviral strategies [84].

Several studies have used ribavirin, an antiviral drug, to treat CHPV. Ribavirin effectively inhibits viral replication in human cells [85]. Vaccination remains the most effective approach against viral infections [86]. Traditional vaccines are both costly and time-intensive, with low efficacy. A multi-epitope vaccine has the potential to overcome the shortcomings of current vaccines by integrating reverse vaccinology and immunoinformatics methodologies. Due to the rise of antibiotic-resistant pathogens and the constraints of conventional vaccines, it is essential to highlight innovative approaches such as multi-epitope vaccines [87,88].

Several studies have employed reverse vaccinology techniques to develop multi-epitope vaccines targeting CHPV. Pavitrakar *et al*. utilized epitopes derived from M, N, P, and G proteins for the construction of a multi-epitope vaccine [89]. Banik *et al*. explored M, N, and G proteins for epitope prediction in another study [90]. The internal proteins (N, P, M, L) of rhabdoviruses are highly conserved compared to the G protein. Their inclusion in vaccine design reduces vulnerability to viral escape through antigenic drift [91] and induces broad CD8^+^ and CD4^+^ responses due to their richness in T-cell epitopes presented via both MHC class I and II pathways [92,93]. However, most epitope prediction pipelines primarily focus on surface proteins, such as G protein. It controls how cells connect to receptors and how pH changes cause fusion. Neutralizing antibodies bind to structural epitopes on G, blocking the virus from entering [94,95]. Antibodies that target internal proteins do not easily attach to intact virus particles or stop the virus from entering cells because these proteins are not present on the surface of the virion [94]. All licensed rhabdovirus vaccines, including rabies vaccines, depend on the G protein [96]. This bias comes from the traditional focus on creating humoral immunity, especially in preventative immunizations where neutralization by antibodies is the most important way to protect people.

RNA viruses have high mutation rates, assisting in rapid evolution and environmental adaption, ultimately maintaining equilibrium with their host [97]. Infected populations have a high mutation rate in the RNA virus genome, which allows the virus to elude the immune system and adapt. As a result, in order to develop a vaccine that is effective against a wide range of viral strains, viral protein sequences from multiple strains must be aligned to gain conserved sequences for selecting efficient epitopes. In this study, we considered the conserved regions of G protein among the 26 pathogenic strains for epitope prediction; and a multi-epitope vaccine against CHPV was developed using optimal CTL, HTL, and linear B-cell epitopes obtained from the conserved regions of G protein, together with appropriate linkers and adjuvant (RS-09 peptide). This study presents, for the first time, the combination of these epitopes with the adjuvant peptide RS-09 to develop a multi-epitope vaccine against the CHPV. This combination is necessary for the vaccination strategy, as neutralizing antibodies are critical for pathogen defense, while helper T cells are pivotal for sustaining CTL response activation and facilitating prolonged antibody production [98].

The findings indicated that the HTL and CTL epitopes of the multi-epitope vaccine demonstrate broad population coverage. The vaccine candidate consists of 214 amino acids, demonstrating favorable antigenicity and solubility while lacking allergenic and toxic properties. The vaccine’s molecular weight was ascertained to be 22.79 kDa; vaccines with molecular weights below 110 kDa may serve as viable candidates due to their superior ease of cloning and production in expression systems compared to larger proteins [99]. The engineered vaccine possesses a theoretical isoelectric of 8.45, signifying its alkaline characteristics and a marginally elevated presence of positive residues. Our vaccine candidate exhibits a half-life of 4.4 hours in mammalian reticulocytes, more than 20 hours in yeast, and more than 10 hours in *E. coli*, demonstrating its stability in cellular contexts. The vaccine’s instability index was calculated to be 36.29; typically, an instability index below 40 is considered stable, and lower indexes produce better results [100]. The vaccine’s aliphatic index was determined to be 83.18, indicating its stability throughout a wide range of temperatures [101]. The negative GRAVY value (−0.062) of the vaccine suggests that the protein possesses hydrophilic characteristics, showing a propensity for beneficial interactions with adjacent water molecules [102].

We modeled and optimized the vaccine’s three-dimensional structure. The analysis of the chosen refined model revealed that 94.2% of the amino acids are located in the most favored regions of the Ramachandran plot, signifying a high-quality stereochemical configuration. The ProSA computed a Z-score of −2.77 for the selectively refined, indicating an appropriate quantity for native proteins.

Docking studies are essential for understanding the molecular interactions that mediate immune responses. By initiating the preliminary recognition phases of the immune system, signaling pathways are activated, hence facilitating the formation of a vigorous immunological response. To boost the immunological response, vaccine adjuvants mostly stimulate TLRs. Understanding how vaccine components interact with specific TLRs can improve vaccine efficacy, adjust immune responses, and aid in the creation of tailored vaccines [103]. CHPV regulates TLR4, causing proinflammatory cytokines and nitric oxide (NO) to be secreted in mouse monocyte-macrophage cells [104]. TLR4 is expressed in human immune cells including monocytes, macrophages, granulocytes, and immature dendritic cells [105]. This study utilized RS-09 peptide (TLR4 agonist) as an adjuvant, employing TLR4 as the receptor for molecular docking. The docking results indicate the vaccine’s capacity to elicit an initial proinflammatory response through the TLR4 receptor, subsequently leading to the development of adaptive immune response cells [106].

The MD simulation results validated the affinity and stability of vaccine binding to TLR4 under settings akin to the physiological milieu. The net energy of the docked complex, as assessed by both MM/PBSA and MM/GBSA techniques, has substantial negative values, signifying that the vaccine-TLR4 complex demonstrates stability and optimal interactions within the cellular milieu [107]. Simulated inoculation with the multi-epitope vaccine resulted in significant increases in both IgM and IgG levels, indicating that the vaccine can successfully create immune memory. This is especially important since the generation of memory B-cells, as well as the fast antibody response upon re-exposure to the antigen, are required for long-term protection and pathogen clearance [108]. The capacity of vaccine designs to induce an antiviral state was evidenced by the elevated levels of IFN-γ and IL-2 [109]. IFN-γ is crucial for viral clearance and the activation of the host immune response [110]. IL-2 is a multifunctional cytokine that promotes the growth, proliferation, and differentiation of T-cells, while significantly enhancing B-cell functionality [111].

The vaccine sequence underwent backtranslation and codon optimization to enhance codon usage for gene expression across various hosts, substituting rare or suboptimal codons in the DNA to reduce the likelihood of translational errors that could impact vaccine immunogenicity and stability. The CAI reached a peak value of 1.0 [112], and the GC content of the optimized codon was 58%, which is within the ideal range of 30–70% [113].

According to a computational examination of the vaccine design, this candidate is expected to be effective against CHPV. The computational design of vaccines facilitates the evaluation of their efficacy using bioinformatics methods, hence enhancing their probability of success in preclinical and clinical trials. Nonetheless, a multi-epitope vaccine possesses certain limitations; specifically, a notable constraint is that most epitope prediction tools inadequately account for the necessity to identify appropriate antigen processing sites, which are crucial for the accurate prediction and presentation of anticipated epitopes. The composition of antigen processing pathways differs according to proinflammatory signals and varies among distinct cell types; thus, present predictive algorithms may be inadequate for assessing the processing efficacy of viral antigens in an infected target cell [114]. While computational techniques provide valuable early insights, it is important to note that bioinformatics predictions are simply the first stage of vaccine development. To move the existing framework closer to practical applicability, numerous critical stages, such as *in vitro* and *in vivo* assays, are planned for the next stage. *In vitro* assays, such the enzyme-linked immunosorbent spot (ELISpot) and intracellular cytokine staining (ICS), can validate T-cell activation by the secretion of IFN-γ and IL-2 [115–117]. The enzyme-linked immunosorbent assay (ELISA) can confirm the generation of antigen-specific and functional antibody responses [118,119]. Research on animals continue to be crucial for assessing immunogenicity, safety, and protective effectiveness. Murine models, particularly HLA-transgenic strains, aid in the evaluation of epitope-specific T-cell responses [120,121], while larger animal models, such as non-human primates, provide more reliable data about immunological durability and protection [122]. The vaccine production procedure is the most difficult and risky in the industry and must adhere to Good Manufacturing Practices (GMPs). Biological products originate from cells, tissues, or microorganisms and exhibit the intrinsic diversity characteristic of living things [123]. Because of potential variation across batches, physicochemical testing procedures make it difficult to describe the active compounds. As a result, particular steps are required to assure consistent product quality [124].

## Conclusion

CHPV has become a notable etiology of acute encephalitis in India, particularly in pediatric populations. Currently, there is no universal vaccine available for CHPV. This study presents the development of a novel multi-epitope vaccine candidate against CHPV via immunoinformatic approaches. Our vaccine candidate comprises an RS-09 peptide (adjuvant), 11 CTL epitopes, 2 HTL epitopes, and 1 linear B-cell epitope. The designed vaccine exhibited a stable physicochemical profile, excellent water solubility, antigenicity, and allergy-free properties. This vaccine can interact stably with TLR4 and elicit a significant and long-lasting immunological response in the patient. Although the current work using *in silico* approaches revealed promising results, *in vitro* and *in vivo* immunological studies are needed to evaluate the potency of the proposed vaccine.

## Supporting information

S1 Data Multiple sequence alignment. (PDF)

S1 Table Predicted CTL epitopes from the GP with percentile rank ≤ 1. (DOCX)

S2 Table Predicted HTL epitopes from the GP with percentile rank ≤ 1. (DOCX)

S3 Table Predicted linear B-cell epitopes from the GP. (DOCX)

S4 Table Population coverage of the chosen CTL epitopes across 16 continents. (DOCX)

S5 Table Population coverage of the chosen HTL epitopes across 16 continents. (DOCX)

S6 Table Population coverage of the chosen CTL and HTL epitopes across 16 continents. (DOCX)

S7 Table The list of predicted docked complexes, along with their energy score and members. (DOCX)

S8 Table List of residue pairs involved in the establishment of H-bonds between vaccine and protein in the docked complex. (DOCX)
