# Supplementary material for: Rational design of multi-epitope vaccine for Chandipura virus using an immunoinformatics approach
Source: PLoS One. 2025 Oct 23;20(10):e0335147. doi: 10.1371/journal.pone.0335147 (PMC12548892; doi:10.1371/journal.pone.0335147)
Supplement: S1 Data — (PDF) [file pone.0335147.s001.pdf]

CLUSTAL O(1.2.4) multiple sequence alignment

```
ASK84898.1          MKSSVTIGVMLIISFINPSYSSLSIAFPENTKLDWKPVTKNTRYCPMGGEWFLEPGLQEE    60
AAU81939.1          MTSSVTISVVLLISFITPSYSSLSIAFPENTKLDWKPVTKNTRYCPMGGEWFLEPGLQEE    60
ADO63668.1          MTSSVTISVILLISFITPSYSSLSIAFPENTKLDWKPVTRNTRYCPMGGEWFLEPGLQEE    60
YP_007641380.1      MTSSVTISVILLISFIAPSYSSLSIAFPENTKLDWKPVTKNTRYCPMGGEWFLEPGLQEE    60
ATB17675.1          MTSSVTISVILLISFITPSYSSLSIAFPENTKLDWKPVTKNTRYCPMGGEWFLEPGLQEE    60
ATB17676.1          MTSSVTISVILLISFITPSYSSLSIAFPENTKLDWKPVTKNTRYCPMGGEWFLEPGLQEE    60
ATB17674.1          MTSSVTISVILLISFITPSYSSLSIAFPENTKLDWKPVTKNTRYCPMGGEWFLEPGLQEE    60
ADO63663.1          MTSSVIISVILLISFITPSYSSLSIAFPENTKLDWKPVTKNTRYCPMGGEWFLEPGLQEE    60
ADO51773.1          MTSSVIISVILLISFITPSYSSLSIAFPENTKLDWKPVTKNTRYCPMGGEWFLEPGLQEE    60
P13180.2            MTSSVTISVVLLISFITPLYSYLSIAFPENTKLDWKPVTKNTRYCPMGGEWFLEPGLQEE    60
AHA42510.1          MTSSVTISVILLISFITPLYSYLSIAFPENTKLDWKPVTKSTRYCPMGGEWFLEPGLQEE    60
AHA42515.1          MTSSVTISVILLISFITPLYSYLSIAFPENTKLDWKPVTKSTRYCPMGGEWFLEPGLQEE    60
AHA42520.1          MTSSVTISVILLISFITPLYSYLSIAFPENTKLDWKPVTKSTRYCPMGGEWFLEPGLQEE    60
WCC64994.1          MLSFMTLYLLAAASVIYPVHSSLSIAFPESTKLDWKPVTKNTRYCPMGGEWFLEPSLQEE    60
WCC64984.1          MLSSMTLCLLAAASVIYPVYSSLSIAFPESTKLDWKPVTKNTRYCPMGGEWFLEPGLQEE    60
WCC64999.1          MLSSMTLCLLAAASMIYPVYSSLSIAFPESTKLDWKPVTKNTRYCPMGGEWFLEPGLQEE    60
WCC64989.1          MLSSITLCLLAAASMIYPVYSSLSIAFPESTKLDWKPVTKNTRYCPMGGEWFLEPGLQEE    60
AED98387.1          MLSQVTLQAFVVMCLIYRAYSSLSIAFPESTKLDWKPVTKNTRYCPMGGEWFLEPGLQEE    60
QNS83649.1          MLSQVTLQAFVVMCLIYRAYSSLSIAFPESTKLDWKPVTKNTRYCPMGGEWFLEPGLQEE    60
QNS83624.1          MLSQVTLQAFVVMCLIYRAYSSLSIAFPESTKLDWKPVTKNTRYCPMGGEWFLEPGLQEE    60
QNS83644.1          MLSQVTLQAFVIVCLIYRAYSSLSIAFPESTKLDWKPVTKNTRYCPMGGEWFLEPGLQEE    60
QNS83619.1          MLSQVTLQAFVVMCLIYRAYSSLSIAFPESTKLDWKPVTKNTRYCPMGGEWFLEPGLQEE    60
QNS83609.1          MLSQVALQTFVIMCLIYRAYSSLSIAFPESTKLDWKPVTKNTRYCPMGGEWFLEPGLQEE    60
QNS83659.1          MLSQVTFQLLVVVCLIYRAYSSLSIAFPESTKLDWKPVTKNTRYCPMGGEWFLEPGLQEE    60
AED98392.1          MLSQVTFQLLVVGCLICQAYSSLSIAFPESTKLDWKPVTKNTRYCPMGGEWFLDPGLQEE    60
QNS83654.1          MLSRVTFQLLVIGCLICQAYSSLSIAFPESTKLDWKPVTKNTRYCPMGGEWFLEPGLQEE    60
                    * * : :  .   ..*   :* *******.*********:.************:*.****

ASK84898.1          SFLSSTPIGATPSKSDGFLCHAAKWVTTCDFRWYGPKYITHSIHNIKPTRSDCDTALASY    120
```

```
AAU81939.1          SFLSSTPIGATPSKSDGFLCHAAKWVTTCDFRWYGPKYITHSIHNIKPTRSDCDTALASY        120
ADO63668.1          SFLSSTPIGATPSKSDGFLCHAAKWVTTCDFRWYGPKYITHSIHNIKPTRSDCDTALASY        120
YP_007641380.1      SFLSSTPIGATPSKSDGFLCHAAKWVTTCDFRWYGPKYITHSIHNIKPTRSDCDTALASY        120
ATB17675.1          SFLSSTPIGATPSKSDGFLCHAAKWVTTCDFRWYGPKYITHSIHNIKPTRSDCDTALASY        120
ATB17676.1          SFLSSTPIGATPSKSDGFLCHAAKWVTTCDFRWYGPKYITHSIHNIKPTRSDCDTALASY        120
ATB17674.1          SFLSSTPIGATPSKSDGFLCHAAKWVTTCDFRWYGPKYITHSIHNIKPTRSDCDTALASY        120
ADO63663.1          SFLSSTPIGATPSKSDGFLCHAAKWVTTCDFRWYGPKYITHSIHNIKPTRSDCDTALASY        120
ADO51773.1          SFLSSTPIGATPSKSDGFLCHAAKWVTTCDFRWYGPKYITHSIHNIKPTRSDCDTALASY        120
P13180.2            SFLSSTPIGATPSKSDGFLCHAAKWVTTCDFRWYGPKYITHSIHNIKPTRSDCDTALASY        120
AHA42510.1          SFLSSTPIGATPSKSDGFLCHAAKWVTTCDFRWYGPKYITHSIHNIKPTRSDCDTALASY        120
AHA42515.1          SFLSSTPIGATPSKSDGFLCHAAKWVTTCDFRWYGPKYITHSIHNIKPTRSDCDTALASY        120
AHA42520.1          SFLSSTPIGATPSKSDGFLCHAAKWVTTCDFRWYGPKYITHSIHNIKPTRSDCDTALASY        120
WCC64994.1          SFISSTPIGATPSKSDGFLCHAAKWVTTCDFRWYGPKYITHSIHNIKPTRSDCDTALASY        120
WCC64984.1          SFLSSTPIGATPSKSDGFLCHAAKWVTTCDFRWYGPKYITHSIHNIKPTRSDCDTALASY        120
WCC64999.1          SFISSTPIGATPSKSDGFLCHAAKWVTTCDFRWYGPKYITHSIHNIKPTRSDCDTALASY        120
WCC64989.1          SFLSSTPIGATPSKSDGFLCHAAKWVTTCDFRWYGPKYITHSIHNIKPTRSDCDTALASY        120
AED98387.1          SFLSSTPIGATPSKSDGFLCHAAKWVTTCDFRWYGPKYITHSIHNIKPTRSDCDSALASY        120
QNS83649.1          SFLSSTPIGATPSKSDGFLCHAAKWVTTCDFRWYGPKYITHSIHNIKPTRSDCDSALASY        120
QNS83624.1          SFLSSTPIGATPSKSDGFLCHAAKWVTTCDFRWYGPKYITHSIHNIKPTRSDCDSALASY        120
QNS83644.1          SFLSSTPIGATPSKSDGFLCHAAKWVTTCDFRWYGPKYITHSIHNIKPTRSDCDSALASY        120
QNS83619.1          SFLSSTPIGATPSKSDGFLCHAAKWVTTCDFRWYGPKYITHSIHNIKPTRSDCDSALASY        120
QNS83609.1          SFLSSTPIGATPSKSDGFLCHAAKWVTTCDFRWYGPKYITHSIHNIKPTRSDCDSALASY        120
QNS83659.1          SFPSSTPIGATPSKSDGFLCHAAKWVTTCDFRWYGPKYITHSIHNIKPTRSDCDSALASY        120
AED98392.1          SFPSSTPIGATPSKSDGFLCHAAKWVTTCDFRWYGPKYITHSIHNIKPTRSDCDSALASY        120
QNS83654.1          SFPSSTPIGATPSKSDGFLCHAAKWVTTCDFRWYGPKYITHSIHNIKPTRSDCDSALASY        120
                    ** ***************************************************:*****

ASK84898.1          KSGTLVSPGFPPESCGYASVTDSEFLVIMITPHHVGVDDYRGHWVDPLFVGGECDQSYCD        180
AAU81939.1          KSGTLVSPGFPPESCGYASVTDSEFLVIMITPHHVGVDDYRGHWVDPLFVGGECDQSYCD        180
ADO63668.1          KSGTLVSPGFPPESCGYASVTDSEFLVIMITPHHVGVDDYRGHWVDPLFVGGECDQSYCD        180
YP_007641380.1      KSGTLVSPGFPPESCGYASVTDSEFLVIMITPHHVGVDDYRGHWVDPLFVGGECDQSYCD        180
ATB17675.1          KSGTLVSPGFPPESCGYASVTDSEFLVIMITPHHVGVDDYRGHWVDPLFVGGECDQSYCD        180
```

```
ATB17676.1          KSGTLVSPGFPPESCGYASVTDSEFLVIMITPHHVGVDDYRGHWVDPLFVGGECDQSYCD        180
ATB17674.1          KSGTLVSPGFPPESCGYASVTDSEFLVIMITPHHVGVDDYRGHWVDPLFVGGECDQSYCD        180
ADO63663.1          KSGTLVSPGFPPESCGYASVTDSEFLVIMITPHHVGVDDYRGHWVDPLFVGGECDQSYCD        180
ADO51773.1          KSGTLVSPGFPPESCGYASVTDSEFLVIMITPHHVGVDDYRGHWVDPLFVGGECDQSYCD        180
P13180.2            KSGTLVSLGFPPESCGYASVTDSEFLVIMITPHHVGVDDYRGHWVDPLFVGGECDQSYCD        180
AHA42510.1          KSGTLVSPGFPPESCGYASVTDSEFLVIMITPHHVGVDDYRGHWVDPLFVGGECDQSYCD        180
AHA42515.1          KSGTLVSPGFPPESCGYASVTDSEFLVIMITPHHVGVDDYRGHWVDPLFVGGECDQSYCD        180
AHA42520.1          KSGTLVSPGFPPESCGYASVTDSEFLVIMITPHHVGVDDYRGHWVDPLFVGGECDQSYCD        180
WCC64994.1          KAGTLVNPGFPPDSCGYATVTDSEFLVIMITPHHVGVDDYRGHWIDPLFVGGECDQSYCD        180
WCC64984.1          KAGTLVNPGFPPDSCGYATVTDSEFLVIMITPHHVGVDDYRGHWVDPLFVGGECDQSYCD        180
WCC64999.1          KAGTLVNPGFPPDSCGYATVTDSEFLVIMITPHHVGVDDYRGHWVDPLFVGGECDQSYCD        180
WCC64989.1          KAGTLVNPGFPPDSCGYATVTDSEFLVVMITPHHVGVDDYRGHWVDPLFVGGECDQSYCD        180
AED98387.1          KSGTLINPGFPPDSCGYASVTDSEFLVIMITPHHVGVDDYRGHWVDPLFVGGECDQSYCD        180
QNS83649.1          KSGTLINPGFPPDSCGYASVTDSEFLVIMITPHHVGVDDYRGHWVDPLFVGGECDQSYCD        180
QNS83624.1          KSGTLINPGFPPDSCGYASVTDSEFLVIMITPHHVGVDDYRGHWVDPLFVGGECDQSYCD        180
QNS83644.1          KSGTLINPGFPPDSCGYASVTDSEFLVIMITPHHVGVDDYRGHWVDPLFVGGECDQSYCD        180
QNS83619.1          KSGTLINPGFPPDSCGYASVTDSEFLVVMITPHHVGVDDYRGHWVDPLFVGGECDQSYCD        180
QNS83609.1          KSGTLINPGFPPDSCGYASVTDSEFLVVMITPHHVGVDDYRGHWVDPLFVGGECDQSYCD        180
QNS83659.1          KSGTLINPGFPPDSCGYASVTDSEFLVIMITPHHVGVDDYRGHWVDPLFVGGECDQSYCD        180
AED98392.1          KSGTLINPGFPPDSCGYASVTDSEFLVIMITPHHVGVDDYRGHWVDPLFVGGECDQSYCD        180
QNS83654.1          KSGTLINPGFPPDSCGYASVTDSEFLVIMITPHHVGVDDYRGHWVDPLFVGGECDQSYCD        180
                    *:***:. ****:*****:********:****************:***************

ASK84898.1          TIHNSSVWIPADSTKKNICGQSFTPLTVTVAYDKTKEIAAGGIVFKSKYHSHMEGARTCR        240
AAU81939.1          TIHNSSVWIPADQTKKNICGQSFTPLTVTVAYVKTKEIAAGAIVFKSKYHSHMEGARTCR        240
ADO63668.1          TIHNSSVWIPADQTKKNICGQSFTPLTVTVAYDKTKEIAAGAIVFKSKYHSHMEGARTCR        240
YP_007641380.1      TIHNSSVWIPADQTKKNICGQSFTPLTVTVAYDKTKEIAAGAIVFKSKYHSHMEGARTCR        240
ATB17675.1          TIHNSSVWIPADQTKKNICGQSFTPLTVTVAYDKTKEIAAGAIVFKSKYHSHMEGARTCR        240
ATB17676.1          TIHNSSVWIPADQTKKNICGQSFTPLTVTVAYDKTKEIAAGALVFKSKYHSHMEGARTCR        240
ATB17674.1          TIHNSSVWIPADQTKKNICGQSFTPLTVTVAYDKTKEIAAGSIVFKSKYHSHMEGARTCR        240
ADO63663.1          TIHNSSVWIPADQTKKNICGQSFTPLTVTVAYDKTKEIAAGAIVFKSKYHSHMEGARTCR        240
ADO51773.1          TIHNSSVWIPADQTKKNICGQSFTPLTVTVAYDKTKEIAAGAIVFKSKYHSHMEGARTCR        240
```

```
P13180.2          TIHNSSVWIPADQTKKNICGQSFTPLTVTVAYDKTKEIAAGGIVFKSKYHSHMEGARTCR    240
AHA42510.1        TIHNSSVWIPADQTKKNICGQSFTPLTVTVAYDKTKEIAAGGIVFKSKYHSHMEGARTCR    240
AHA42515.1        TIHNSSVWIPADQTKKNICGQSFTPLTVTVAYDKTKEIAAGGIVFKSKYHSHMEGARTCR    240
AHA42520.1        TIHNSSVWIPADQTKKNICGQSFTPLTVTVAYDKTKEIAAGGIVFKSKYHSHMEGARTCR    240
WCC64994.1        TIHNSSVWMPSDTTKKNICGQSFIPLTVTVAYDKTKDISAGALVFKSKYHSHMEGARTCR    240
WCC64984.1        TIHNSSVWMPSDTTKKNICGQSFTPLTVTVAYDKTKEISAGALVFKSKYHSHMEGARTCR    240
WCC64999.1        TIHNSSVWMPSDTTKKNICGQSFIPLTVTVAYDKTKDISAGALVFKSKYHSHMEGARTCR    240
WCC64989.1        TIHNSSVWMPSDTTKKNICGQSFIPLTVTVAYDKTKDISAGALVFKSKYHSHMEGARTCR    240
AED98387.1        TIHNSSVWIPADTTKKNICGQSFTPLTVTVAYDKTKDISAGALVFKSKYHSHMEGARTCR    240
QNS83649.1        TIHNSSVWIPADTTKKNICGQSFTPLTVTVAYDKTKDISAGALVFKSKYHSHMEGARTCR    240
QNS83624.1        TIHNSSVWIPADTTKKNICGQSFTPLTVTVAYNRTKDISAGALVFKSKYHSHMEGARTCR    240
QNS83644.1        TIHNSSVWIPADTTKKNICGQSFTPLTVTVAYDKTKDISAGALVFKSKYHSHMEGARTCR    240
QNS83619.1        TIHNSSVWIPADTTKKNICGQSFTPLTVTVAYDKTKDISAGALVFKSKYHSHMEGARTCR    240
QNS83609.1        TIHNSSVWIPADTTKKNICGQSFTPLTVTVAYDKTKDISAGALVFKSKYHSHMEGARTCR    240
QNS83659.1        TIHNSSVWIPADTKKKNICGQSFTPLTVTVAYDKTKDISAGALVFKSKYHSHMEGARTCR    240
AED98392.1        TIHNSSVWIPADTKKKNICGQSFTPLTVTVAYDKTKDITAGALVFKSKYHSHMEGARTCR    240
QNS83654.1        TIHNSSVWIPADTKKKNICGQSFTPLTVTVAYDKTKDITAGALVFKSKYHSHMEGARTCR    240
                  ********:*:* .********* ******** :**:*:**.:*****************

ASK84898.1        LSYCGRNGIKFPNGEWVSLDVKTKIQEKPLLPLFKECPAGTEVRSTLQSDGAQVLTSEIQ    300
AAU81939.1        LSYCGRNGIKFPNGEWVSLDVKTKIQEKPLLPLFKECPAGTEVRSTLQSDGAQVLTSEIQ    300
ADO63668.1        LSYCGRNGIKFPNGEWVSLDVKTKIQEKPLLPLFKECPAGTEVRSTLQSDGAQVLTSEIQ    300
YP_007641380.1    LSYCGRNGIKFPNGEWVSLDVKTKIQEKPLLPLFKECPAGTEVRSTLQSDGAQVLTSEIQ    300
ATB17675.1        LSYCGRNGIKFPNGEWVSLDVKTKIQEKPLLPLFKECPAGTEVRSTLQSDGAQVLTSEIQ    300
ATB17676.1        LSYCGRNGIKFPNGEWVSLDVKTKIQEKPLLPLFKECPAGTEVRSTLQSDGAQVLTSEIQ    300
ATB17674.1        LSYCGRNGIKFPNGEWVSLDVKTKIQEKPLLPLFKECPAGTEVRSTLQSDGAQVLTSEIQ    300
ADO63663.1        LSYCGRNGIKFPNGEWVSLDVKTKIQEKPLLPLFKECPAGTEVRSTLQSDGAQVLTSEIQ    300
ADO51773.1        LSYCGRNGIKFPNGEWVSLDVKTKIQEKPLLPLFKECPAGTEVRSTLQSDGAQVLTSEIQ    300
P13180.2          LSYCGRNGIKFPNGEWVSLDVKTRIQEKHLLPLFKECPAGTEVRSTLQSDGAQVLTSEIQ    300
AHA42510.1        LSYCGRNGIKFPNGEWVSLDVKTKIQEKPLLPLFKECPAGTEVRSTLQSDGAQVLTPEIQ    300
AHA42515.1        LSYCGRNGIKFPNGEWVSLDVKTKIQEKPLLPLFKECPAGTEVRSTLQSDGAQVLTSEIQ    300
AHA42520.1        LSYCGRNGIKFPNGEWVSLDVKTKIQEKPLLPLFKECPAGTEVRSTLQSDGAQVLTSEIQ    300
```

```
WCC64994.1          LTYCGRTGIKFPNGEWVSLDIKTKIQEKQLLPLFKECPAGTEVRSTLQSDGAQVLTSEIQ        300
WCC64984.1          LTYCGRTGIKFPNGEWVSLDTKTKIQEKQLLPLFKECPAGTEVRSTLQSDGAQVLTSEIQ        300
WCC64999.1          LTYCGRTGIKFPNGEWVSLDTKTKIQEKQLLPLFKECPAGTEVRSTLQSDGAQVLTSEIQ        300
WCC64989.1          LTYCGRTGIKFPNGEWVSLDTKTKIQEKQLLPLFKECPAGTEVRSTLQSDGAQVLTSEIQ        300
AED98387.1          LTYCGRTGIKFPNGEWVSLDIKTKIQDKQLLPLFKECPAGTEVRSTLQSDGAQVLTSEIQ        300
QNS83649.1          LTYCGRTGIKFPNGEWVSLDIKTKIQDKQLLHLFKECPAGTEVRSTLQSDGAQVLTSEIQ        300
QNS83624.1          LTYCGRTGIKFPNGEWVSLDTKTKIQEKQLLPLFKECPAGTEVRSTLQSDGAQVLTSEIQ        300
QNS83644.1          LTYCGRTGIKFPNGEWVSLDIKTKIQEKQLLPLFKECPAGTEVRSTLQSDGAQVLTSEIQ        300
QNS83619.1          LTYCGRTGIKFPNGEWVSLDTKTKIQEKQLLPLFKECPAGTEVRSTLQSDGAQVLTSEIQ        300
QNS83609.1          LTYCGRTGIKFPNGEWVSLDTKTKIQEKQLLPLFKECPAGTEVRSTLQSDGAQVLTSEIQ        300
QNS83659.1          LTYCGRTGIKFPNGEWVSLDVKTKIQEKQLLPLFKECPAGTEVRSTLQSDGAQVLTSEIQ        300
AED98392.1          LTYCGRTGIKFPNGEWVSLDVKTKIQEKQLLPLFKECPAGTEVRSTLQSDGAQVLTSEIQ        300
QNS83654.1          LTYCGRTGIKFPNGEWVSLDVKTKIQEKQLLPLFKECPAGTEVRSTLQSDGAQVLTSEIQ        300
                    *:****.************* **:**:* ** ************************ ***

ASK84898.1          RILDYSLCQNTWDKVERKEPLSPLDLSYLASKSPGKGLAYTVINGTLAFAHTRYVRMWID        360
AAU81939.1          RILDYSLCQNTWDKVERKEPLSPLDLSYLASKSPGKGLAYTVINGTLSFAHTRYVRMWID        360
ADO63668.1          RILDYSLCQNTWDKVERKEPLSPLDLSYLASKSPGKGLAYTVINGTLSFAHTRYVRMWID        360
YP_007641380.1      RILDYSLCQNTWDKVERKEPLSPLDLSYLASKSPGKGLAYTVINGTLSFAHTRYVRMWID        360
ATB17675.1          RILDYSLCQNTWDKVERKEPLSPLDLSYLASKSPGKGLAYTVINGTLSFAHTRYVRMWID        360
ATB17676.1          RILDYSLCQNTWDKVERKEPLSPLDLSYLASKSPGKGLAYTVINGTLSFAHTRYVRMWID        360
ATB17674.1          RILDYSLCQNTWDKVERKEPLSPLDLSYLASKSPGKGLAYTVINGTLSFAHTRYVRMWID        360
ADO63663.1          RILDYSLCQNTWDKVERKEPLSPLDLSYLASKSPGKGLAYTVINGTLSFAHTRYVRMWID        360
ADO51773.1          RILDYSLCQNTWDKVERKEPLSPLDLSYLASKSPGKGLAYTVINGTLSFAHTRYVRMWID        360
P13180.2            RILDYSLCQNTWDKVERKEPLSPLDLSYLASKSPGKGLAYTVINGTLSFAHTRYVRMWID        360
AHA42510.1          RILDYSLCQNTWDKVERKEPLSPLDLSYLASKSPGKGLAYTVINGTLSFAHTRYVRMWID        360
AHA42515.1          RILDYSLCQNTWDKVERKEPLSPLDLSYLASKSPGKGLAYTVINGTLSFAHTRYVRMWID        360
AHA42520.1          RILDYSLCQNTWDKVERKEPLSPLDLSHLASKSPGKGLAYTVINGTLSFAHTRYVRMWID        360
WCC64994.1          RLLDYSLCQNTWEKVDRKEPLSPLDLSYLASKSPGKGLAYTVINGTLSFAHTRYVRMWID        360
WCC64984.1          RLLDYSLCQNTWEKVDRKEPLSPLDLSYLASKSPGKGLAYTVINGTLSFAHTRYVRMWID        360
WCC64999.1          RLLDYSLCQNTWEKVDRKEPLSPLDLSYLASKSPGKGLAYTVINGTLSFAHTRYVRMWID        360
WCC64989.1          RLLDYSLCQNTWEKVDRKEPLSPLDLSYLASKSPGKGLAYTVINGTLSFAHTRYVRMWID        360
```

```
AED98387.1          RLLDYSLCQNTWEKVDRKEPLSPLDLSYLASKSPGKGLAYTVINGTLSFAHTRYVRMWID        360
QNS83649.1          RLLDYSLCQNTWEKVDRKEPLSPLDLSYLASKSPGKGLAYTVINGTLSFAHTRYVRMWID        360
QNS83624.1          RLLDYSLCQNTWEKVDRKEPLSPLDLSYLASKSPGKSLAYTVINGTLSFAHTRYVRMWID        360
QNS83644.1          RLLDYSLCQNTWEKVDRKEPLSPLDLSYLASKSPGKGLAYTVINGTLSFAHTRYVRMWID        360
QNS83619.1          RLLDYSLCQNTWEKVDRKEPLSPLDLSYLASKSPGKGLAYTVINGTLSFAHTRYVRMWID        360
QNS83609.1          RLLDYSLCQNTWEKVDRKEPLSPLDLSYLASKSPGKGLAYTVINGTLSFAHTRYVRMWID        360
QNS83659.1          RLLDYSLCQNTWEKVDRKEPLSPLDLSYLASKSPGKGLAYTVINGTLSFAHTRYVRMWID        360
AED98392.1          RLLDYSLCQNTWEKVDRKEPLSPLDLSYLASKSPGKGLAYTVINGTLSFAHTRYVRMWID        360
QNS83654.1          RLLDYSLCQNTWEKVDRKEPLSPLDLSYLASKSPGKGLAYTVINGTLSFAHTRYVRMWID        360
                    *:**********:**:***********:********.**********:************

ASK84898.1          GPVLKELKGKRESPSGISSDIWTQWFKYGDMEIGPNGLLKTAGGYKFPWHLIGMGIVDNE        420
AAU81939.1          GPVLKEMKGKRESPSGISSDIWTQWFKYGDMEIGPNGLLKTAGGYKFPWHLIGMGIVDNE        420
ADO63668.1          GPVLKEMKGKRESPSGISSDIWTQWFKYGDMEIGPNGLLKTAGGYKFPWHLIGMGIVDNE        420
YP_007641380.1      GPVLKEMKGKRESPSGISSDIWTQWFKYGDMEIGPNGLLKTAGGYKFPWHLIGMGIVDNE        420
ATB17675.1          GPVLKEMKGKRESPSGISSDIWTQWFKYGDMEIGPNGLLKTAGGYKFPWHLIGMGIIDNE        420
ATB17676.1          GPVLKEMKGKRESPSGISSDIWTQWFKYGDMEIGPNGLLKTAGGYKFPWHLIGMGIVDNE        420
ATB17674.1          GPVLKEMKGKRESPSGISSDIWTQWFKYGDMEIGPNGLLKTAGGYKFPWHLIGMGIVDNE        420
ADO63663.1          GPVLKEMKGKRESPSGISSDIWTQWFKYGDMEIGPNGLLKTAGGYKFPWHLIGMGIVDNE        420
ADO51773.1          GPVLKEMKGKRESPSGISSDIWTQWFKYGDMEIGPNGLLKTAGGYKFPWHLIGMGIVDNE        420
P13180.2            GPVLKEPKGKRESPSGISSDIWTQWFKYGDMEIGPNGLLKTAGGYKFPWHLIGMGIVDNE        420
AHA42510.1          GPVLKEPKGKRESPSGISSDIWTQWFKYGDMEIGPNGLLKTAGGYKFPWHLIGMGIVDNE        420
AHA42515.1          GPVLKEPKGKRESPSGISSDIWTQWFKYGDMEIGPNGLLKTAGGYKFPWHLIGMGIVDNE        420
AHA42520.1          GPVLKEPKGKRESPSGISSDIWTQWFKYGDMEIGPNGLLKTAGGYKFPWHLIGMGIVDNE        420
WCC64994.1          GPLLKELKGKRESASGIATEIWNQWFKYGDMEIGPNGLLKTKSGYKFPWHLIGMGIVDNE        420
WCC64984.1          GPVLNELKGKRESASGIATEIWNQWFKYGDMEIGPNGLLKTKNGYKFPWHLIGMGIVDNE        420
WCC64999.1          GPLLKELKGKRESASGIATEIWNQWFKYGDMEIGPNGLLKTKNGYKFPWHLIGMGIVDNE        420
WCC64989.1          GPLLKELKGKRESASGIATEIWNQWFKYGDMEIGPNGLLKTKSGYKFPWHLIGMGIVDNE        420
AED98387.1          GPVLKELKGKRESASGIASDIWTQWFKYGDMEIGPNGLLKTKSGYKFPWHLIGMGIVDNE        420
QNS83649.1          GPVLKELKGKRESASGIASDIWTQWFKYGDMEIGPNGLLKTKSGYKFPWHLIGMGIVDNE        420
QNS83624.1          GPVLKELKGKRESASGIASDIWTQWFKYGDMEIGPNGLLKTKSGYKFPWHLIGMGIVDNE        420
QNS83644.1          GPVLKELKGKRESASGIASDIWTQWFKYGDMEIGPNGLLKTKSGYKFPWHLIGMGIVDNE        420
```

```
QNS83619.1          GPVLKELKGKRESASGIASDIWTQWFKYGDMEIGPNGLLKTKSGYKFPWHLIGMGIVDNE        420
QNS83609.1          GPVLKELKGKRESASGIASDIWTQWFKYGDMEIGPNGLLKTKSGYKFPWHLIGMGIVDNE        420
QNS83659.1          GPVLKELKGKRESASGIASDIWTQWFKYGDMEIGPNGLLKTKSGYKFPWHLIGMGIVDNE        420
AED98392.1          GPVLKELKGKRESASGIASDIWTQWFKYGDMEIGPNGLLKTKSGYKFPWHLIGMGIVDNE        420
QNS83654.1          GPVLKELKGKRESASGIASDIWTQWFKYGDMEIGPNGLLKTKSGYKFPWHLIGMGIVDNE        420
                    **:*:* ****** ***:::**.****************** .*************:***

ASK84898.1          LHELSEANPLDHPQLPHAQSIADDSEEIFFGDTGVSKNPVELVTGWFTSWKESLAAGVVL        480
AAU81939.1          LHELSEANPLDHPQLPHAQSIADDSEEIFFGDTGVSKNPVELVTGWFTSWKESLAAGVVL        480
ADO63668.1          LHEVSEANPLDHPQLPHAQSIADDSEEIFFGDTGVSKNPVELVTGWFTSWKESLAAGVVL        480
YP_007641380.1      LHELSEANPLDHPQLPHAQSIADDSEEIFFGDTGVSKNPVELVTGWFTSWKESLAAGVVL        480
ATB17675.1          LHELSEANPLDHPQLPHAQSIADDSEEIFFGDTGVSKNPVELVTGWFTSWKESLAAGVVL        480
ATB17676.1          LHELSEANPLDHPQLPHAQSIADDSEEIFFGDTGVSKNPVELVTGWFTSWKESLAAGVVL        480
ATB17674.1          LHELSEANPLDHPQLPHAQSIADDSEEIFFGDTGVSKNPVELVTGWFTSWKESLAAGVVL        480
ADO63663.1          LHELSEANPLDHPQLPHAQSIADDSEEIFFGDTGVSKNPVELVTGWFTSWKESLAAGVVL        480
ADO51773.1          LHELSEANPLDHPQLPHAQSIADDSEEIFFGDTGVSKNPVELVTGWFTSWKESLAAGVVL        480
P13180.2            LHELSEANPLDHPQLPHAQSIADDSEEIFFGDTGVSKNPVELVTGWFTSWKESLAAGVVL        480
AHA42510.1          LHELSEANPLDHPQLPHAQSIADDSEEIFFGDTGVSKNPVELVTGWFTSWKESLAAGVVL        480
AHA42515.1          LHELSEANPLDHPQLPHAQSIADDSEEIFFGDTGVSKNPVELVTGWFTSWKESLAAGVVL        480
AHA42520.1          LHELSEANPLDHPQLPHAQSIADDSEEIFFGDTGVSKNPVELVTGWFTSWKESLAAGVVL        480
WCC64994.1          LHELSEANPLDHPQLPHAQSIADDSEEIFFGDTGVSKNPVELVTGWFTSWKESLAAGVVL        480
WCC64984.1          LHELSEANPLDHPQLPHAQSIADDSEEIFFGDTGVSKNPVELVTGWFTSWKESLAAGVVL        480
WCC64999.1          LHELSEANPLDHPQLPHAQSIADDSEEIFFGDTGVSKNPVELVTGWFTSWKESLAAGVVL        480
WCC64989.1          LHELSEANPLDHPQLPHAQSIADDSEEIFFGDTGVSKNPVELVTGWFTSWKESLAAGVVL        480
AED98387.1          LHELSEANPLDHPQLPHAQSIADDSEEIFFGDTGVSKNPVELVTGWFTSWKESLAAGVVL        480
QNS83649.1          LHELSEANPLDHPQLPHAQSIADDSEEIFFGDTGVSKNPVELVTGWFTSWKESLAAGVVL        480
QNS83624.1          LHELSEANPLDHPQLPHAQSIADDSEEIFFGDTGVSKNPVELVTGWFTSWKESLAAGVVL        480
QNS83644.1          LHELSEANPLDHPQLPHAQSIADDSEEIFFGDTGVSKNPVELVTGWFTSWKESLAAGVVL        480
QNS83619.1          LHELSEANPLDHPQLPHAQSIADDSEEIFFGDTGVSKNPVELVTGWFTSWKESLAAGVVL        480
QNS83609.1          LHELSEANPLDHPQLPHAQSIADDSEEIFFGDTGVSKNPVELVTGWFTSWKESLAAGVVL        480
QNS83659.1          LHELSEANPLDHPQLPHAQSIADDSEEIFFGDTGVSKNPVELVTGWFTSWKESLAAGVVL        480
AED98392.1          LHELSEANPLDHPQLPHAQSIADDSEEIFFGDTGVSKNPVELVTGWFTSWKESLAAGVVL        480
```

```
QNS83654.1          LHEWSEANPLDHPQLPHAHSIADDSEEIFFGDTGVSKNPVELVTGWFTSWKESLAAGVVL        480
                    *** **************:*****************************************

ASK84898.1          ILVVVLIYGVLRCFPVLCTTCRKPKWKKGVERSDSFEMRIFKPNNMRARV  530
AAU81939.1          ILVVVLIYGVLRCFPVLCTTCRKPKWKKGVERSDSFEMRIFKPNNMRARV  530
ADO63668.1          ILVVVLIYGVLRCFPVLCTTCRKPKWKKGVERSDSFEMRIFKPNNMRARV  530
YP_007641380.1      ILVVVLIYGVLRCFPVLCTTCRKPKWKKGVERSDSFEMRIFKPNNMRARV  530
ATB17675.1          ILVVVLIYGVLRCFPVLCTTCRKPKWKKGVERSDSFEMRIFKPNNMRARV  530
ATB17676.1          ILVVVLIYGVLRCFPVLCTTCRKPKWKKGVERSDSFEMRIFKPNNMRARV  530
ATB17674.1          ILVVVLIYGVLRCFPVLCTTCRKPKWKKGVERSDSFEMRIFKPNNMRARV  530
ADO63663.1          ILVVVLIYGVLRCFPVLCTTCRKPKWKKGVERSDSFEMRIFKPNNMRARV  530
ADO51773.1          ILVVVLIYGVLRCFPVLCTTCRRPKWKKGVERSDSFEMRIFKPNNMRARV  530
P13180.2            ILVVVLIYGVLRCFPVLCTTCRKPKWKKGVERSDSFEMRIFKPNNMRARV  530
AHA42510.1          ILVVVLIYGVLRCFPVLCTTCRKPKWKKGVERSDSFEMRIFKPNNMRARV  530
AHA42515.1          ILVVVLIYGVLRCFPVLCTTCRKPKWKKGVERSDSFEMRIFKPNNMRARV  530
AHA42520.1          ILVVVLIYGVLRCFPVLCTTCRKPKWKKGVERSDSFEMRIFKPNNMRARV  530
WCC64994.1          ILTVVLIYGVLRCFPVLCMPCRRTKWKKGVERSDSFEMRVFKPNNMRARV  530
WCC64984.1          ILTVVLIYGVLRCFPVLCMPCRRTKWKKGVKRSDSFEMRVFKPNNMRARV  530
WCC64999.1          ILTVVLIYGVLRCFPVLCMPCRRTKWKKGVERSDSFEMRIFKPNNMRARV  530
WCC64989.1          ILTVVLIYGVLRCFPVLFMPCRRTKWKKGVERSDSFEMRIFKPNNMRARV  530
AED98387.1          ILTVVLIYGVLRCFPVLCMPCRKTKWKKEVERSDSFEMRIFKPNNMRARV  530
QNS83649.1          ILTVVLIYGVLRCFPVLCMPCRKTKWKKEVERSDSFEMRIFKPNNMRARV  530
QNS83624.1          ILTVVLIYGVLRCFPVLCMPCRKTKWKKGVERSDSFEMRIFKPNNMRARV  530
QNS83644.1          ILTVVLIYGVLRCFPVLCMPCRKTKWKKGVERSDSFEMRIFKPNNMRARV  530
QNS83619.1          ILTVVLIYGVLRCFPVLCMPCRKTKWKKGVERSDSFEMRIFKPNNMRARV  530
QNS83609.1          ILTVVLIYGVLRCFPVLCMPCRKTKWKKGVERSDSFEMRIFKPNNMRARV  530
QNS83659.1          ILTVVLIYGVLRCFPVLCIPCKKPKWKKGVERSDSFEMRIFKPNNMRARV  530
AED98392.1          ILTVVLIYGVLRCFPVLCMPCKKPKWKKGVERSDSFEMRIFKPNNMRARV  530
QNS83654.1          ILTVVLIYGVLRCFPVLCMPCKKPKWKKGVERSDSFEMRIFKPNNMRARV  530
                    **.**************   *:: **** *:********:**********
```
