# Supplementary material for: Rational design of multi-epitope vaccine for Chandipura virus using an immunoinformatics approach
Source: PLoS One. 2025 Oct 23;20(10):e0335147. doi: 10.1371/journal.pone.0335147 (PMC12548892; doi:10.1371/journal.pone.0335147)
Supplement: S1 Table — (DOCX) [file pone.0335147.s002.docx]

**Table S1**

Predicted CTL epitopes from the GP with percentile rank ≤ 1. The CTL epitopes that have been selected are shown in bold.

| **CTL epitope** | **Allele** | **Length** | **Vaxijen score** | **Allergenicity** | **Toxicity** |
| --- | --- | --- | --- | --- | --- |
| AHTRYVRMW | HLA-B*58:01, HLA-A*26:01, HLA-B*35:01, HLA-B*51:01, HLA-B*08:01 | 9 | -0.2781 | Allergen | Non-Toxic |
| ATPSKSDGF | HLA-A*26:01, HLA-B*07:02 | 9 | 0.0784 | Non-Allergen | Non-Toxic |
| AYTVINGTL | HLA-A*24:02, HLA-A*23:01 | 9 | 0.3274 | Allergen | Toxic |
| CDTIHNSSVW | HLA-B*57:01, HLA-B*58:01 | 10 | -0.2184 | Non-Allergen | Non-Toxic |
| CPMGGEWFL | HLA-B*53:01, HLA-B*35:01 | 9 | 0.3319 | Allergen | Non-Toxic |
| DTIHNSSVW | HLA-B*53:01, HLA-A*26:01, HLA-B*58:01 HLA-B*57:01, HLA-B*35:01 | 9 | -0.3822 | Non-Allergen | Non-Toxic |
| DYSLCQNTW | HLA-A*24:02, HLA-A*23:01, HLA-B*53:01 | 9 | -0.5291 | Non-Allergen | Non-Toxic |
| **EANPLDHPQL** | **HLA-A*68:02, HLA-B*51:01, HLA-B*53:01** | **10** | **0.7144** | **Non-Allergen** | **Non-Toxic** |
| **EIFFGDTGV** | **HLA-A*68:02, HLA-A*26:01** | **9** | **1.0213** | **Non-Allergen** | **Non-Toxic** |
| EIGPNGLLK | HLA-A*68:01, HLA-A*03:01, HLA-A*11:01 | 9 | 0.5872 | Allergen | Non-Toxic |
| ELVTGWFTSW | HLA-A*26:01, HLA-B*57:01, HLA-A*32:01, HLA-B*53:01 | 10 | -0.4106 | Allergen | Non-Toxic |
| **ESLAAGVVLI** | **HLA-A*68:02** | **10** | **0.6009** | **Non-Allergen** | **Non-Toxic** |
| FAHTRYVRMW | HLA-B*57:01, HLA-B*53:01, HLA-B*58:01 | 10 | -0.0363 | Allergen | Non-Toxic |
| FPNGEWVSL | HLA-B*35:01, HLA-B*08:01, HLA-B*51:01, HLA-B*07:02, HLA-B*53:01 | 9 | 0.7309 | Allergen | Non-Toxic |
| **FPWHLIGMGI** | **HLA-B*51:01, HLA-B*53:01** | **10** | **1.4709** | **Non-Allergen** | **Non-Toxic** |
| FVGGECDQSY | HLA-A*01:01, HLA-A*26:01 | 10 | 0.1432 | Allergen | Non-Toxic |
| GFLCHAAKW | HLA-A*23:01, HLA-A*24:02 | 9 | 0.2322 | Allergen | Non-Toxic |
| GIKFPNGEW | HLA-A*32:01, HLA-B*57:01 | 9 | 0.2344 | Non-Allergen | Toxic |
| GPKYITHSI | HLA-B*07:02, HLA-B*51:01, HLA-B*08:01, HLA-B*53:01 | 9 | 0.0433 | Allergen | Non-Toxic |
| GVDDYRGHW | HLA-B*58:01, HLA-A*32:01, HLA-B*57:01, HLA-B*53:01 | 9 | 1.3094 | Allergen | Toxic |
| GVSKNPVEL | HLA-A*02:06, HLA-A*02:03 | 9 | 0.3480 | Non-Allergen | Non-Toxic |
| **GYKFPWHLI** | **HLA-A*24:02, HLA-A*23:01** | **9** | **0.4265** | **Non-Allergen** | **Non-Toxic** |
| HSIHNIKPTR | HLA-A*68:01, HLA-A*31:01, HLA-A*33:01 | 10 | 0.9749 | Allergen | Toxic |
| IADDSEEIF | HLA-B*53:01, HLA-B*58:01, HLA-A*01:01, HLA-B*35:01 | 9 | -0.3213 | Non-Allergen | Non-Toxic |
| IADDSEEIFF | HLA-A*01:01, HLA-B*53:01 | 10 | 0.0327 | Non-Allergen | Non-Toxic |
| ITHSIHNIK | HLA-A*30:01, HLA-A*11:01, HLA-A*03:01, HLA-A*68:01, HLA-A*31:01 | 9 | 0.5568 | Allergen | Toxic |
| **KECPAGTEV** | **HLA-B*40:01, HLA-B*44:02, HLA-B*44:03** | **9** | **0.9987** | **Non-Allergen** | **Non-Toxic** |
| KFPNGEWVSL | HLA-B*07:02, HLA-A*24:02, HLA-A*23:01 | 10 | 0.3182 | Allergen | Non-Toxic |
| KFPWHLIGM | HLA-A*24:02, HLA-A*23:01 | 9 | 1.0107 | Allergen | Non-Toxic |
| KNPVELVTGW | HLA-B*53:01, HLA-B*57:01, HLA-B*58:01 | 10 | 0.1702 | Allergen | Non-Toxic |
| **KWVTTCDFRW** | **HLA-B*58:01, HLA-B*57:01, HLA-A*23:01, HLA-A*24:02** | **10** | **1.0820** | **Non-Allergen** | **Non-Toxic** |
| KYITHSIHNI | HLA-A*24:02, HLA-A*23:01 | 10 | 0.0876 | Non-Allergen | Toxic |
| LDYSLCQNTW | HLA-B*44:02, HLA-A*23:01, HLA-B*44:03, HLA-A*24:02 | 10 | -0.4296 | Allergen | Non-Toxic |
| LIYGVLRCF | HLA-A*32:01, HLA-A*26:01, HLA-B*15:01 | 9 | -0.2853 | Allergen | Non-Toxic |
| LQSDGAQVL | HLA-B*15:01, HLA-B*40:01, HLA-A*02:06 | 9 | -0.5963 | Allergen | Non-Toxic |
| LVTGWFTSW | HLA-A*32:01, HLA-A*26:01, HLA-B*53:01, HLA-B*58:01, HLA-B*57:01 | 9 | -0.4595 | Allergen | Toxic |
| **MEIGPNGLL** | **HLA-B*40:01, HLA-B*44:02, HLA-B*44:03** | **9** | **1.0283** | **Non-Allergen** | **Non-Toxic** |
| MEIGPNGLLK | HLA-A*03:01, HLA-A*11:01 | 10 | 0.7889 | Allergen | Non-Toxic |
| MITPHHVGV | HLA-A*68:02, HLA-A*02:03, HLA-A*02:06 HLA-A*02:01, HLA-B*08:01 | 9 | 1.1731 | Allergen | Toxic |
| NPVELVTGW | HLA-B*53:01, HLA-A*26:01, HLA-B*51:01, HLA-B*35:01 | 9 | 0.5322 | Allergen | Non-Toxic |
| NPVELVTGWF | HLA-B*53:01, HLA-B*35:01 | 10 | 0.3596 | Allergen | Non-Toxic |
| RWYGPKYITH | HLA-A*30:02, HLA-A*31:01, HLA-A*30:01 | 10 | -1.0357 | Allergen | Non-Toxic |
| RYCPMGGEW | HLA-A*24:02, HLA-A*23:01, HLA-A*32:01, HLA-B*58:01, HLA-B*57:01 | 9 | 1.7905 | Allergen | Toxic |
| RYCPMGGEWF | HLA-A*24:02, HLA-A*23:01 | 10 | 1.1968 | Allergen | Non-Toxic |
| SIADDSEEI | HLA-A*02:06, HLA-A*68:02, HLA-A*32:01, HLA-A*02:06, HLA-A*02:01, HLA-A*02:03, HLA-A*02:01, HLA-A*02:03, HLA-A*68:02 | 9 | 0.1518 | Non-Allergen | Non-Toxic |
| SIHNIKPTR | HLA-A*31:01, HLA-A*33:01, HLA-A*68:01, HLA-A*30:01, HLA-A*11:01, HLA-A*03:01 | 9 | 1.2080 | Non-Allergen | Toxic |
| **SLAAGVVLIL** | **HLA-A*02:03, HLA-A*02:01** | **10** | **0.5275** | **Non-Allergen** | **Non-Toxic** |
| STLQSDGAQV | HLA-A*02:03, HLA-A*68:02 | 10 | -0.3178 | Allergen | Non-Toxic |
| **STPIGATPSK** | **HLA-A*11:01, HLA-A*03:01, HLA-A*30:01, HLA-A*68:01** | **10** | **0.9458** | **Non-Allergen** | **Non-Toxic** |
| TLQSDGAQV | HLA-A*02:03, HLA-A*02:06, HLA-A*02:01 | 9 | -0.3717 | Allergen | Non-Toxic |
| TPHHVGVDDY | HLA-B*35:01, HLA-B*53:01 | 10 | 0.9370 | Non-Allergen | Toxic |
| **TPIGATPSK** | **HLA-A*68:01, HLA-B*07:02, HLA-B*53:01** | **9** | **1.1768** | **Non-Allergen** | **Non-Toxic** |
| TRYCPMGGEW | HLA-A*23:01, HLA-A*24:02 | 10 | 1.6415 | Allergen | Toxic |
| VGVDDYRGHW | HLA-B*57:01, HLA-B*58:01 | 10 | 1.4086 | Non-Allergen | Toxic |
| **VSKNPVELV** | **HLA-A*30:01, HLA-A*68:02** | **9** | **0.5395** | **Non-Allergen** | **Non-Toxic** |
| VTGWFTSWK | HLA-A*11:01, HLA-A*03:01 | 9 | -0.4720 | Allergen | Toxic |
| VVLIYGVLR | HLA-A*31:01, HLA-A*68:01, HLA-A*33:01 | 9 | -0.0181 | Non-Allergen | Non-Toxic |
| WVTTCDFRW | HLA-B*58:01, HLA-B*57:01, HLA-B*53:01 | 9 | 0.9221 | Allergen | Non-Toxic |
| YCPMGGEWFL | HLA-B*53:01, HLA-B*35:01 | 10 | 0.5640 | Allergen | Non-Toxic |
| YGPKYITHSI | HLA-B*51:01, HLA-B*07:02, HLA-B*08:01 | 10 | 0.2502 | Allergen | Non-Toxic |
| YITHSIHNI | HLA-A*02:06, HLA-A*02:03, HLA-A*68:02, HLA-A*02:01 | 9 | 0.3353 | Allergen | Toxic |
| YITHSIHNIK | HLA-A*68:01, HLA-A*11:01 | 10 | 0.5810 | Non-Allergen | Toxic |
