# Supplementary material for: Rational design of multi-epitope vaccine for Chandipura virus using an immunoinformatics approach
Source: PLoS One. 2025 Oct 23;20(10):e0335147. doi: 10.1371/journal.pone.0335147 (PMC12548892; doi:10.1371/journal.pone.0335147)
Supplement: S2 Table — (DOCX) [file pone.0335147.s003.docx]

**Table S2**

Predicted HTL epitopes from the GP with percentile rank ≤ 1. The HTL epitopes that have been selected are shown in bold.

| **HTL epitope** | **Allele** | **Vaxijen score** | **Allergenicity** | **Toxicity** | **FN-γ inducer** | **IL-4 inducer** |
| --- | --- | --- | --- | --- | --- | --- |
| **DSEEIFFGDTGVSKN** | **HLA-DQA1*01:01/DQB1*05:01** | **0.6905** | **Non-Allergen** | **Non-Toxic** | **+** | **+** |
| **GPKYITHSIHNIKPT** | **HLA-DQA1*04:01/DQB1*04:02, HLA-DRB1*07:01, HLA-DPA1*02:01/DPB1*01:01, HLA-DPA1*02:01/DPB1*05:01, HLA-DRB1*09:01, HLA-DPA1*01:03/DPB1*02:01, HLA-DPA1*03:01/DPB1*04:02, HLA-DRB1*13:02** | **0.7254** | **Non-Allergen** | **Non-Toxic** | **+** | **+** |
| **PKYITHSIHNIKPTR** | **HLA-DQA1*04:01/DQB1*04:02** | **0.6855** | **Non-Allergen** | **Non-Toxic** | **+** | **+** |
| SSTPIGATPSKSDGF | HLA-DQA1*05:01/DQB1*03:01 | 0.5700 | Non-Allergen | Non-Toxic | - | - |
| STPIGATPSKSDGFL | HLA-DQA1*05:01/DQB1*03:01 | 0.5229 | Non-Allergen | Non-Toxic | - | + |
| TGWFTSWKESLAAGV | HLA-DQA1*04:01/DQB1*04:02 | 0.0595 | Allergen | Non-Toxic | - | + |
| VTGWFTSWKESLAAG | HLA-DQA1*04:01/DQB1*04:02 | -0.0624 | Allergen | Non-Toxic | - | + |
| WYGPKYITHSIHNIK | HLA-DQA1*04:01/DQB1*04:02 | 0.2234 | Non-Allergen | Non-Toxic | + | + |
| YGPKYITHSIHNIKP | HLA-DQA1*04:01/DQB1*04:02, HLA-DRB1*07:01, HLA-DPA1*02:01/DPB1*01:01, HLA-DPA1*03:01/DPB1*04:02, HLA-DPA1*02:01/DPB1*14:01, HLA-DPA1*02:01/DPB1*05:01, HLA-DRB1*09:01, HLA-DPA1*01:03/DPB1*02:01, HLA-DRB1*13:02 | 0.6185 | Allergen | Non-Toxic | + | + |
