# Supplementary material for: Rational design of multi-epitope vaccine for Chandipura virus using an immunoinformatics approach
Source: PLoS One. 2025 Oct 23;20(10):e0335147. doi: 10.1371/journal.pone.0335147 (PMC12548892; doi:10.1371/journal.pone.0335147)
Supplement: S3 Table — (DOCX) [file pone.0335147.s004.docx]

**Table S3**

Predicted linear B-cell epitopes from the GP. The linear B-cell epitope that have been selected are shown in bold.

| **Linear B-cell epitope** | **Length** | **Vaxijen score** | **Allergenicity** | **Toxicity** |
| --- | --- | --- | --- | --- |
| ATPSKSDGF | 9 | 0.0784 | Non-Allergen | Non-Toxic |
| DFRWYGPKYITHSIHNIKPTR | 21 | 0.5844 | Allergen | Non-Toxic |
| **DSEEIFFGDTGVSKNPVEL** | **19** | **0.6358** | **Non-Allergen** | **Non-Toxic** |
| GGECDQSYCDTIHNS | 15 | -0.0248 | Non-Allergen | Non-Toxic |
| GVDDYR | 6 | 2.4141 | Allergen | Non-Toxic |
| KYHSHMEGART | 11 | 0.1071 | Allergen | Non-Toxic |
| PAGTEVRSTLQSDGA | 15 | 0.4411 | Allergen | Non-Toxic |
| YGDMEIGPNG | 10 | 1.5890 | Allergen | Non-Toxic |
