# Supplementary material for: Rational design of multi-epitope vaccine for Chandipura virus using an immunoinformatics approach
Source: PLoS One. 2025 Oct 23;20(10):e0335147. doi: 10.1371/journal.pone.0335147 (PMC12548892; doi:10.1371/journal.pone.0335147)
Supplement: S4 Table — (DOCX) [file pone.0335147.s005.docx]

**Table S4**

Population coverage of the chosen CTL epitopes across 16 continents.

| **population/area** | **CTL epitopes** | | |
| --- | --- | --- | --- |
|  | **coverage^a^** | **average_hit^b^** | **pc90^c^** |
| Central Africa | 74.45% | 2.0 | 0.39 |
| Central America | 6.44% | 0.15 | 0.11 |
| East Africa | 78.27% | 2.22 | 0.46 |
| East Asia | 87.43% | 2.34 | 0.8 |
| Europe | 94.16% | 2.54 | 1.19 |
| North Africa | 84.76% | 2.43 | 0.66 |
| North America | 93.38% | 2.79 | 1.18 |
| Northeast Asia | 87.36% | 1.97 | 0.79 |
| Oceania | 88.43% | 2.1 | 0.86 |
| South Africa | 79.34% | 2.23 | 0.48 |
| South America | 74.8% | 1.8 | 0.4 |
| South Asia | 78.11% | 1.72 | 0.46 |
| Southeast Asia | 90.33% | 2.32 | 1.02 |
| Southwest Asia | 76.74% | 1.75 | 0.43 |
| West Africa | 82.27% | 2.37 | 0.56 |
| West Indies | 91.98% | 2.89 | 1.13 |
| World | 91.16% | 2.39 | 1.05 |
| **Average** | **79.97** | **2.12** | **0.7** |
| **Standard deviation** | **19.48** | **0.59** | **0.32** |

^a^ projected population coverage
^b^ average number of epitope hits / HLA combinations recognized by the population
^c^ minimum number of epitope hits / HLA combinations recognized by 90% of the population
