# Supplementary material for: Rational design of multi-epitope vaccine for Chandipura virus using an immunoinformatics approach
Source: PLoS One. 2025 Oct 23;20(10):e0335147. doi: 10.1371/journal.pone.0335147 (PMC12548892; doi:10.1371/journal.pone.0335147)
Supplement: S5 Table — (DOCX) [file pone.0335147.s006.docx]

**Table S5**

Population coverage of the chosen HTL epitopes across 16 continents.

| **population/area** | **HTL epitopes** | | |
| --- | --- | --- | --- |
|  | **coverage^a^** | **average_hit^b^** | **pc90^c^** |
| Central Africa | 99.78% | 3.19 | 2.18 |
| Central America | 96.23% | 1.84 | 1.12 |
| East Africa | 99.5% | 3.36 | 2.1 |
| East Asia | 93.08% | 1.96 | 1.1 |
| Europe | 99.76% | 2.3 | 1.4 |
| North Africa | 78.41% | 1.29 | 0.46 |
| North America | 99.98% | 2.9 | 1.83 |
| Northeast Asia | 95.57% | 2.1 | 1.22 |
| Oceania | 97.35% | 2.25 | 1.36 |
| South Africa | 7.65% | 0.08 | 0.11 |
| South America | 99.37% | 3.4 | 2.02 |
| South Asia | 98.66% | 2.15 | 1.32 |
| Southeast Asia | 77.08% | 1.08 | 0.44 |
| Southwest Asia | 69.02% | 0.96 | 0.32 |
| West Africa | 99.56% | 3.05 | 2.06 |
| West Indies | 52.44% | 1.02 | 0.21 |
| World | 97.29% | 2.46 | 1.36 |
| **Average** | **85.93** | **2.08** | **1.21** |
| **Standard deviation** | **23.61** | **0.92** | **0.68** |

^a^ projected population coverage
^b^ average number of epitope hits / HLA combinations recognized by the population
^c^ minimum number of epitope hits / HLA combinations recognized by 90% of the population
