# Supplementary material for: Rational design of multi-epitope vaccine for Chandipura virus using an immunoinformatics approach
Source: PLoS One. 2025 Oct 23;20(10):e0335147. doi: 10.1371/journal.pone.0335147 (PMC12548892; doi:10.1371/journal.pone.0335147)
Supplement: S6 Table — (DOCX) [file pone.0335147.s007.docx]

**Table S6**

Population coverage of the chosen CTL and HTL epitopes across 16 continents.

| **population/area** | **Combined CTL and HTL epitopes** | | |
| --- | --- | --- | --- |
|  | **coverage^a^** | **average_hit^b^** | **pc90^c^** |
| Central Africa | 99.94% | 5.19 | 3.08 |
| Central America | 96.47% | 1.98 | 1.14 |
| East Africa | 99.89% | 5.58 | 3.16 |
| East Asia | 99.13% | 4.3 | 2.38 |
| Europe | 99.99% | 4.84 | 3.06 |
| North Africa | 96.71% | 3.72 | 1.66 |
| North America | 100.0% | 5.69 | 3.47 |
| Northeast Asia | 99.44% | 4.07 | 2.37 |
| Oceania | 99.69% | 4.35 | 2.67 |
| South Africa | 80.92% | 2.3 | 0.52 |
| South America | 99.84% | 5.2 | 2.99 |
| South Asia | 99.71% | 3.87 | 2.22 |
| Southeast Asia | 97.78% | 3.4 | 1.8 |
| Southwest Asia | 92.79% | 2.71 | 1.15 |
| West Africa | 99.92% | 5.41 | 3.16 |
| West Indies | 96.19% | 3.9 | 1.69 |
| World | 99.76% | 4.85 | 2.88 |
| **Average** | **97.54** | **4.2** | **2.32** |
| **Standard deviation** | **4.58** | **1.09** | **0.84** |

^a^ projected population coverage
^b^ average number of epitope hits / HLA combinations recognized by the population
^c^ minimum number of epitope hits / HLA combinations recognized by 90% of the population
