# Supplementary material for: Rational design of multi-epitope vaccine for Chandipura virus using an immunoinformatics approach
Source: PLoS One. 2025 Oct 23;20(10):e0335147. doi: 10.1371/journal.pone.0335147 (PMC12548892; doi:10.1371/journal.pone.0335147)
Supplement: S7 Table — (DOCX) [file pone.0335147.s008.docx]

**Table S7**

The list of predicted docked complexes, along with their energy score and members.

| **Cluster** | **Members** | **Representative** | **Weighted Score** |
| --- | --- | --- | --- |
| **0** | 53 | Center | -978.7 |
|  |  | Lowest Energy | -1157.0 |
| **1** | 36 | Center | -1047.6 |
|  |  | Lowest Energy | -1096.0 |
| **2** | 30 | Center | -1057.2 |
|  |  | Lowest Energy | -1231.3 |
| **3** | 25 | Center | -992.9 |
|  |  | Lowest Energy | -1195.4 |
| **4** | 25 | Center | -1005.4 |
|  |  | Lowest Energy | -1107.6 |
| **5** | 24 | Center | -1033.0 |
|  |  | Lowest Energy | -1232.1 |
| **6** | 24 | Center | -1025.2 |
|  |  | Lowest Energy | -1109.2 |
| **7** | 21 | Center | -1076.1 |
|  |  | Lowest Energy | -1076.1 |
| **8** | 19 | Center | -1006.4 |
|  |  | Lowest Energy | -1095.0 |
| **9** | 19 | Center | -993.8 |
|  |  | Lowest Energy | -1129.5 |
| **10** | 18 | Center | -1127.9 |
|  |  | Lowest Energy | -1159.0 |
| **11** | 17 | Center | -1087.8 |
|  |  | Lowest Energy | -1087.8 |
| **12** | 17 | Center | -1082.8 |
|  |  | Lowest Energy | -1109.3 |
| **13** | 17 | Center | -1050.1 |
|  |  | Lowest Energy | -1050.1 |
| **14** | 15 | Center | -985.1 |
|  |  | Lowest Energy | -1130.8 |
| **15** | 15 | Center | -1018.2 |
|  |  | Lowest Energy | -1078.0 |
| **16** | 15 | Center | -1126.5 |
|  |  | Lowest Energy | -1200.7 |
| **17** | 15 | Center | -1063.4 |
|  |  | Lowest Energy | -1117.8 |
| **18** | 13 | Center | -1033.7 |
|  |  | Lowest Energy | -1115.7 |
| **19** | 13 | Center | -979.0 |
|  |  | Lowest Energy | -1037.9 |
| **20** | 13 | Center | -1118.4 |
|  |  | Lowest Energy | -1136.5 |
| **21** | 13 | Center | -1048.4 |
|  |  | Lowest Energy | -1048.4 |
| **22** | 12 | Center | -974.4 |
|  |  | Lowest Energy | -1100.7 |
| **23** | 12 | Center | -1142.5 |
|  |  | Lowest Energy | -1160.5 |
| **24** | 12 | Center | -1060.8 |
|  |  | Lowest Energy | -1060.8 |
| **25** | 12 | Center | -1040.3 |
|  |  | Lowest Energy | -1040.3 |
| **26** | 11 | Center | -1064.3 |
|  |  | Lowest Energy | -1088.0 |
| **27** | 11 | Center | -1000.1 |
|  |  | Lowest Energy | -1144.2 |
| **28** | 11 | Center | -1124.9 |
|  |  | Lowest Energy | -1124.9 |
| **29** | 11 | Center | -1022.4 |
|  |  | Lowest Energy | -1079.3 |
