# Supplementary material for: Rational design of multi-epitope vaccine for Chandipura virus using an immunoinformatics approach
Source: PLoS One. 2025 Oct 23;20(10):e0335147. doi: 10.1371/journal.pone.0335147 (PMC12548892; doi:10.1371/journal.pone.0335147)
Supplement: S8 Table — (DOCX) [file pone.0335147.s009.docx]

**Table S8**

List of residue pairs involved in the establishment of H-bonds between vaccine and protein in the docked complex.

| Vaccine | TLR4 | Distance (Å) |
| --- | --- | --- |
| GLY108 | LYS560 (Chain A) | 2.67 |
| TYR112 | LYS560 (Chain A) | 2.62 |
| TYR99 | ASN531 (Chain B) | 3.03 |
| TYR99 | PHE533 (Chain B) | 2.82 |
| GLU8 | LYS541 (Chain B) | 2.45 |
| ALA97 | LYS560 (Chain B) | 2.63 |
| ALA98 | LYS560 (Chain B) | 2.59 |
| TRP102 | GLU563 (Chain B) | 3.03 |
| LYS12 | HIS566 (Chain B) | 2.85 |
